# *In vitro* Selection and Interaction Studies of a DNA Aptamer Targeting Protein A

**DOI:** 10.1371/journal.pone.0134403

**Published:** 2015-07-29

**Authors:** Regina Stoltenburg, Thomas Schubert, Beate Strehlitz

**Affiliations:** 1 UFZ—Helmholtz Centre for Environmental Research, Department of Soil Ecology, Halle, Germany; 2 2bind GmbH, Regensburg, Germany; 3 UFZ—Helmholtz Centre for Environmental Research, Department Environmental and Biotechnology Centre, Leipzig, Germany; Ben-Gurion University, ISRAEL

## Abstract

A new DNA aptamer targeting Protein A is presented. The aptamer was selected by use of the FluMag-SELEX procedure. The SELEX technology (Systematic Evolution of Ligands by EXponential enrichment) is widely applied as an *in vitro* selection and amplification method to generate target-specific aptamers and exists in various modified variants. FluMag-SELEX is one of them and is characterized by the use of magnetic beads for target immobilization and fluorescently labeled oligonucleotides for monitoring the aptamer selection progress. Structural investigations and sequence truncation experiments of the selected aptamer for Protein A led to the conclusion, that a stem-loop structure at its 5’-end including the 5’-primer binding site is essential for aptamer-target binding. Extensive interaction analyses between aptamer and Protein A were performed by methods like surface plasmon resonance, MicroScale Thermophoresis and bead-based binding assays using fluorescence measurements. The binding of the aptamer to its target was thus investigated in assays with immobilization of one of the binding partners each, and with both binding partners in solution. Affinity constants were determined in the low micromolar to submicromolar range, increasing to the nanomolar range under the assumption of avidity. Protein A provides more than one binding site for the aptamer, which may overlap with the known binding sites for immunoglobulins. The aptamer binds specifically to both native and recombinant Protein A, but not to other immunoglobulin-binding proteins like Protein G and L. Cross specificity to other proteins was not found. The application of the aptamer is directed to Protein A detection or affinity purification. Moreover, whole cells of *Staphylococcus aureus*, presenting Protein A on the cell surface, could also be bound by the aptamer.

## Introduction

Protein A is a cell surface protein of the gram-positive, pathogenic bacterium *Staphylococcus aureus* and exists in both cell wall-bound and secreted forms [1]. *Staph*. *aureus* is a ubiquitous human pathogen causing a range of diseases from minor skin infections to systemic and life-threatening diseases such as pneumonia, meningitis, osteomyelitis, endocarditis, toxic shock syndrome (TSS), bacteremia, and sepsis [2, 3]. It is known as a predominant cause of nosocomial infections. Along with the use of antibiotics for treatment of bacterial infections it became evident that *Staph*. *aureus* is remarkable in its ability to acquire resistance to any antibiotics [4]. Such antibiotic-resistant strains, designated MRSA (methicillin-resistant *Staph*. *aureus*), are not only a risk of the hospital-associated infections, but also cause increasingly community-associated infections, and therefore represent a major public health problem.

The broad range of infections caused by *Staph*. *aureus* is based on a number of virulence factors, with Protein A as one of them [2]. Protein A is well known for its interaction with immunoglobulins [5, 6]. It comprises five highly homologous Ig-binding domains and possesses two distinct Ig-binding activities. Protein A has high affinities to the Fc region of several subclasses of human IgG and of IgG from other mammalian species (as well as weak affinities to human IgM and IgA) and is also able to bind to the Fab region of the Ig heavy chain, especially of the V_H_3 family (e.g., Fab regions of the B-cell receptor) [7, 8]. These features help *Staph*. *aureus* to circumvent the protective immune responses of the host by inhibition of phagocytosis and preventing the production of pathogen-specific antibodies [3]. Moreover, the immunoglobulin binding ability of Protein A is commonly used in biological basic research and immunology. The protein is often recombinant produced in *E*. *coli* and applied as tool for purifying, immobilization and detection of immunoglobulins.

Protein A also represents a very attractive target for aptamer selection to generate specific binding agents applicable as diagnostic tools for detection of pathogenic *Staph*. *aureus* cells, as analytical tools in environmental or food analysis, and in biological basic research for targeting Protein A. Aptamers are special single stranded nucleic acid molecules, which can be used like antibodies. Different from the conventional view on nucleic acids as carrier of genetic information, aptamers are more like globular molecules, and their functionality is based on their complex three-dimensional structure. The intramolecular folding in accordance with the primary sequence of the aptamers enables them to recognize and bind their targets with high affinity and specificity. Such target-specific aptamers are generated by the SELEX technology, an iterative *in vitro* selection and amplification method starting from an oligonucleotide library comprising a large sequence diversity and structural complexity [9, 10]. Since the first publication of aptamers in 1990, they have been selected for a wide variety of different targets from small molecules, like nucleotides, cofactors, or amino acids over peptides, polysaccharides, and proteins to complex structures like whole cells, viruses, and single cell organisms [11, 12].

As a very attractive class of targeting agents, aptamers are in great demand in many fields of application, e. g., in medical and pharmaceutical basic research as well as in clinical diagnostics and therapy. Moreover, aptamers have a very promising potential as molecular recognition elements in a wide range of analytical systems [13]. In this context, there is a growing interest in the development of aptamer based detection assays and sensing systems, which are applicable in environmental, water, or food analytics.

Aptamer publications over the last years reveal that the development of aptamers for pathogenic microbes, especially for bacterial pathogens and their applications become more important. Aptamers were selected for gram-negative and gram-positive bacteria like *E*.*coli*, *Salmonella typhi*, *S*. *typhimurium*, *S*. *enteritidis*, *Campylobacter jejuni*, *Francisella tularensis*, *Shigella dysenteriae*, *Mycobacterium tuberculosis*, *Bacillus anthracis*, *Streptococcus pyogenes*, *Listeria monocytogenes*, or *P*. *aeruginosa* with the aim of targeting the whole bacterial cells for analytical or diagnostic applications [14]. Several studies also focus on the development of aptamers for *Staphylococcus aureus* using whole cells as selection target [15–18]. None of these identified the specific target molecule on the cell surface of the bacteria. In contrast, Maeng et al. and Han et al. described the selection of RNA aptamers for teichoic acid as a component of the gram-positive cell wall of *Staph*. *aureus* [19, 20]. Baumstummler et al. reported the generation of SOMAmers (special modified DNA aptamers) for a set of cell surface associated proteins with Protein A as one of them. They could show the functionality of these different aptamers for the specific capture and detection of *Staph*. *aureus* [21]. Friedman et al. recently described the use of Protein A as a model target for the direct selection of RNA aptamers from a special 2’-fully modified RNA library [22].

In this work a new DNA aptamer for Protein A selected by the FluMag-SELEX process [23] is described. The focus of the experimental studies was on the interaction analyses between the aptamer and Protein A regarding the affinity/avidity, specificity, and binding site. Different methods like bead-based binding assays, surface plasmon resonance (SPR) based measurements and MicroScale Thermophoresis (MST) were applied. Truncation experiments were performed to narrow down the sequence region of the aptamer essentially for binding to the target. Future studies would expand the present work on analyzing the interaction of the aptamer with whole bacterial cells of *Staph*. *aureus* to demonstrate its utility as detecting agent not only for Protein A but also for the corresponding bacterial pathogen.

## Materials and Methods

### Chemicals

Protein A from *Staphylococcus aureus* (P3838), biotinylated native Protein A (P2165), and recombinant Protein A (P7837, expressed in *E*. *coli*), as well as human serum albumin (HSA, A9511), bovine serum albumin (BSA, A3059), human thrombin (89223 Fluka), and human immunoglobulin G (IgG, I4506) were purchased from Sigma-Aldrich (Germany). Human IgG-Fc fragment (009–0103) and human IgG-Fab fragment (009–0105) were from Rockland Immunochemicals, Inc. (Ireland). Recombinant Protein G and Protein L (Pierce 21193 and 21189, expressed in *E*. *coli*) were purchased from Fisher Scientific (Germany). Superparamagnetic Dynabeads M-270 Streptavidin (Strep-MB) were purchased from Invitrogen/Life Technologies (USA). According to the manufacturer’s instructions, these streptavidin-coated magnetic beads were used for immobilization of biotinylated native Protein A (P2165) as the target protein for aptamer selection to obtain Protein A-modified Strep-MB (Protein A/Strep-MB). PCR components like 10 × reaction buffer, 25 mM MgCl_2_ and HOTFire polymerase were purchased from Solis BioDyne (Estonia). 100 mM stock solutions of dNTPs were from GE Healthcare (Germany).

### SELEX library and primers

The SELEX library BANK-C was synthesized by Microsynth (Switzerland) including a PAGE (polyacrylamide gel electrophoresis) purification step. The library consists of a multitude of different oligonucleotides. Each of them contains a central random region of 40 nt (N) flanked by specific sequences of 18 nt at the 5’- and 3’-end, which function as primer binding sites for the PCR: 5‘-ATACCAGCTTATTCAATT–N_40_ –ACAATCGTAATCAGTTAG-3‘. The following primers were used for amplification of the oligonucleotides during the aptamer selection process and were synthesized by biomers.net (Germany): AP10: 5‘-ATACCAGCTTATTCAATT-3‘, AP60: the modified variant of AP10 with 5’-fluorescein, AP30: 5’-CTAACTGATTACGATTGT-3’, and TER-AP30: the modified variant of AP30 with 5’-poly-dA_20_-HEGL.

### DNA aptamer selection by FluMag-SELEX

The selection of DNA aptamers for Protein A from *Staphylococcus aureus* was carried out using a protocol based on the FluMag-SELEX procedure (S1 Fig) [23]. Each SELEX round was started with the thermal equilibration of the oligonucleotide pool in selection buffer (100 mM NaCl, 20 mM Tris-HCl pH 7.6, 10 mM MgCl_2_, 5 mM KCl, 1 mM CaCl_2_). 2.5 nmol of the SELEX library oligonucleotides in the first round and, in each of the following rounds, the total quantity of selected oligonucleotides from the previous round, respectively, were heated to 90°C for 8 min, immediately cooled, and kept at 4°C for 10 min followed by a short incubation at room temperature. In parallel, an aliquot of 1 × 10^8^ Protein A/Strep-MB was washed three times with selection buffer. Both components, the target modified beads and the pretreated oligonucleotide pool, were taken together to initiate the selection steps in each round. The complete SELEX procedure is described in detail in Supporting Information (S1 File). Some procedure adaptions during the aptamer selection procedure were made to affect the specificity and affinity of the oligonucleotides. From round 7 only two of the four different fractions of target bound oligonucleotides were amplified and processed for the next SELEX round (only the fractions of oligonucleotides eluted by selection buffer from the Protein A/Strep-MB) (S1 File). To remove nonspecific binding oligonucleotides, a negative selection step with unmodified Strep-MB prior to positive selection with Protein A/Strep-MB was introduced in SELEX round 3 and in rounds 7–11. As the SELEX rounds progressed and an enrichment of target bound oligonucleotides could be observed, the stringency was increased by increasing the number, length, and volume of washing steps of the binding complexes.

A fluorescein label was attached to the oligonucleotides from round two onwards during the amplification step in each SELEX round. This enables the quantification of the target bound oligonucleotides after their elution from the Protein A/Strep-MB, which is important in order to assess the aptamer selection progress over several SELEX rounds.

The selected oligonucleotide pool from SELEX round 11 was amplified with unmodified primers and subsequently cloned using the TOPO TA Cloning Kit (Invitrogen/Life Technologies, USA). 96 clones were further analyzed by sequencing and alignment of obtained sequences using the web based tool ClustalW (http://www.ebi.ac.uk/Tools/msa/clustalw2/) [24, 25]. The secondary structure analysis was performed by means of the free-energy minimization algorithm according to Zuker [26] using the internet tool mfold at 21°C with 100 mM [Na^+^] and 10 mM [Mg^++^] (http://mfold.rna.albany.edu/?q=mfold) [27, 28].

### Fluorescence detection

All fluorescence measurements of fluorescein-labeled DNA were performed on a Wallac 1420 Victor^2^ V Multilabel Counter (PerkinElmer, Germany) with excitation at 485 nm and emission at 535 nm (prompt fluorometry, time 1 s, CW-lamp energy 22500). The readings were performed in black 96 microwell plates from NUNC/Thermo Fisher Scientific (Germany) with a sample volume of 100 μL/well. A calibration curve in the range of 0.4–80 pmol/mL of fluorescein-labeled ssDNA prepared from the SELEX library BANK-C was used to calculate the DNA concentration in SELEX samples. In the case of purchased fluorescein-labeled aptamers applied in individual binding assays, a calibration curve of each of them was measured and used for quantification.

### Bead-based binding assay

Individual aptamers were synthesized with a 5’-fluorescein label by Microsynth (Switzerland) including a PAGE purification step. Fluorescein-labeled ssDNA from the SELEX library BANK-C and from the selected aptamer pool was prepared by PCR and PAGE purification as described for the FluMag-SELEX procedure (S1 File). Comparative bead-based binding assays were performed according to the FluMag-SELEX conditions. For each assay, a fresh aliquot of 3 × 10^7^ target modified magnetic beads (Protein A/Strep-MB) was washed three times with 250 μL selection buffer. During the second washing step the beads were incubated at 21°C for 5 min in selection buffer with mild shaking before magnetic separation of the beads. 55 pmol fluorescein-labeled aptamers in 250 μL selection buffer were heated to 90°C for 8 min, immediately cooled, and kept at 4°C for 10 min followed by a short incubation at room temperature before adding it to the washed beads. Incubation was performed at 21°C for 30 min with mild shaking for binding of the aptamers to Protein A/Strep-MB. The unbound aptamers were removed by 3 to 5 washing steps with 250 μL selection buffer and the bound aptamers were then eluted twice by incubating the binding complexes in 250 μL selection buffer at 95°C for 10 min with mild shaking. The amount of aptamers eluted from the beads was determined by fluorescence detection and calculation using a calibration curve.

The bead-based binding assays were also used to determine the affinity (*K*_D_) of the aptamers. Different aptamer concentrations in the range of 13–3550 nM were incubated with a constant number of Protein A/Strep-MB (3 × 10^7^ beads) for binding. The amounts of bound aptamers were then quantified by fluorescence detection. On the basis of these data, a saturation curve was obtained and the dissociation constant *K*_D_ was calculated by non-linear regression analysis using OriginPro 9.0 (OriginLab Corporation, Northampton, MA, USA).

### SPR measurements

The SPR measurements were carried out with a Biacore X100 instrument. The Biotin CAPture Kit (GE Healthcare Europe GmbH, Germany) was used for the interaction studies according to manufacturer’s instructions. It enables reversible capture of biotinylated ligands and includes the sensor chip CAP, the Biotin CAPture Reagent, and a two-component regeneration solution. The basic surface of sensor chip CAP consists of a carboxymethylated dextran matrix modified with a pre-immobilized oligonucleotide. To build up a specific sensor surface, streptavidin conjugated with the complementary oligonucleotide (Biotin CAPture Reagent) is added and hybridized to the surface followed by capture of the biotinylated ligand. To start the interaction analysis (association/dissociation), the analyte sample is subsequently added. After the dissociation phase, the sensor surface is regenerated by disrupting the hybridized oligonucleotides for a new experimental cycle. This means, the complete specific sensor surface has to be rebuilt in every cycle. The level of the Biotin CAPture Reagent and also of the immobilized ligand was checked every time to ensure an intact sensor surface. If the levels decreased significantly, a new sensor chip CAP was used.

5’- or 3’-biotinylated aptamers as well as the biotinylated SELEX library BANK-C and a truncated library BANK-C58 (same as BANK-C, but 58 nt long with a central random region of 22 nt instead of 40 nt) were synthesized by BioSpring GmbH (Germany). After hybridization of the Biotin CAPture Reagent to the basic sensor surface according to manufacturer’s instructions, 1–5 μM biotinylated aptamer in running buffer (same as selection buffer + 0.005% surfactant P20) was injected at a flow rate of 5 μL/min for immobilization. Comparable immobilization levels of commonly 1000–1200 RU were adjusted over the injection time. Reduced levels were applied to specific experiments and are indicated in the result section. The unselected library was immobilized in the reference flow cell by the same way to allow background signal subtraction. The truncated library was used for the same purpose in experiments with truncated aptamer variants. Protein A was serially diluted in running buffer to a concentration range of 10–5000 nM and injected at 21°C at a flow rate of 10 μL/min for 300 s (association phase) followed by a dissociation phase of 300 s. The injection of sample containing 1000 nM Protein A was performed in duplicate or triplicate at different time points within each experiment. All experiments were carried out at least two times. Double referencing of each data set was achieved by use of the reference sensor surface modified with the unselected SELEX library (as described above) and injections of running buffer. Data were processed using the BIAevaluation software (GE Healthcare Europe GmbH, Germany), and OriginPro 9.0 (OriginLab Corporation, Northampton, MA, USA) was used for plotting the steady-state binding from the end of the association phase against analyte concentration.

In addition to the multi-cycle mode to analyze the binding between aptamer and Protein A as described above, the single-cycle mode was also performed. 3’-biotinylated aptamer (0.5 μM in running buffer; flow rate of 5 μL/min) was immobilized on the sensor surface to a level of 516 RU. Protein A solutions of 62, 185, 556, 1667, and 5000 nM were prepared in running buffer and sequentially injected (starting with the lowest concentration) at a flow rate of 30 μL/min for 180 s followed by 180 s dissociation. The last injection was followed by 600 s dissociation and subsequent regeneration of the sensor surface. All measurements of this binding analysis were performed in triplicate. A reference flow cell modified with the unselected library BANK-C and buffer injection were used for double referencing the binding data.

Interaction analyses were also performed using biotinylated native Protein A (42 kDa) as ligand, which was immobilized on the sensor surface. For this purpose 100 nM biotinylated Protein A in running buffer was injected for 180 s at a flow rate of 5 μL/min (ligand level ~600 RU). The reference flow cell was left blank, only Biotin CAPture Reagent was hybridized. 5’-fluorescein-labeled aptamers synthesized by Microsynth (Switzerland) were used as analytes in this case. They were serially diluted in running buffer to a concentration range of 250 nM—8000 nM and injected at 21°C at a flow rate of 10 μL/min for 420 s (association phase) followed by a dissociation phase of 600 s. The injection of sample containing 2000 nM aptamer was performed in duplicate or triplicate at different time points within each experiment. As described above all experiments were done at least two times and under conditions allowing double referencing of the binding data.

### MicroScale Thermophoresis (MST)

The MicroScale Thermophoresis technology enables the quantitative analysis of biomolecular interactions in solution and allows the determination of binding affinities. It uses a fluorescent label or the intrinsic fluorescence of one of the binding partners to monitor the movement of these molecules in a temperature gradient. The thermophoretic mobility of the fluorescent molecules will be changed upon forming of the binding complexes [29].

MST measurements were carried out with a constant concentration of 50 nM 5’-fluorescein-labeled aptamers in combination with a final concentration series of 1.69–55,555 nM recombinant Protein A and 0.33–10,820 nM native Protein A, respectively. For this purpose, the protein was titrated in 16 steps 1:1 starting from the stock solution of 111,110 nM recombinant Protein A or 21,640 nM native Protein A. 10 μL of aptamer stock solution (100 nM) and 10 μL Protein A of each titration step were mixed for binding reactions. The analyses were performed in selection buffer with addition of 0.005% Tween 20 at a temperature of 25°C. All MST measurements were carried out on a Monolith NT.115 (NanoTemper Technologies GmbH, Germany) using standard capillaries. The Laser and LED powers were adjusted to get optimized results in each experiment. The recorded fluorescence was normalized to fraction bound (0 = unbound, 1 = bound), processed using the software KaleidaGraph 4.5.2, and fitted using the *K*_D_ fit formula derived from the law of mass action. For native Protein A, a ligand dependent enhancement effect was detected. Due to this, the raw fluorescence was used for analysis.

## Results and Discussion

### Selection and functional features of aptamer PA#2/8 targeting Protein A

**Fig 1:**
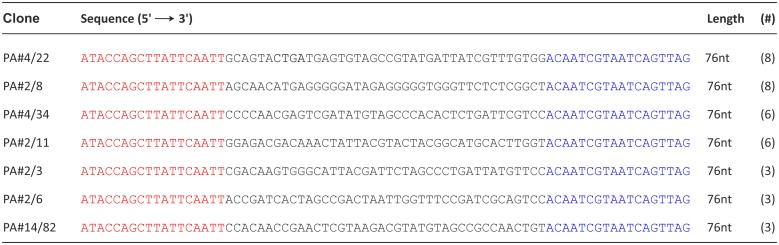
Most abundant aptamer sequences in the selected aptamer pool. Seven groups with 3–8 homologous sequences (#: number of homologous sequences) were identified among the sequenced aptamer clones. The representative aptamer clone of each group is shown. The specific primer binding sites at the 5’- and 3’-end of the aptamer clones are colored in red and blue, respectively.

The selection of DNA aptamers for Protein A as a cell surface protein of *Staphylococcus aureus* was performed using the FluMag-SELEX procedure [23]. This SELEX variant is based on magnetic beads as immobilization matrix for the target molecules (herein Protein A/Strep-MB) and on the utilization of a fluorescence label for quantification of the DNA during the SELEX process (herein 5’-fluorescein modified oligonucleotides) (S1 Fig). After 7 SELEX rounds, a stepwise enrichment of target-bound oligonucleotides was observed for the next 4 selection rounds (S2 Fig). The selection conditions were changed from round 7 onwards concerning the insertion of a negative selection step with unmodified Strep-MB, the elution step, and the stringency of washing steps of the binding complexes in round 9–11 (S2 Fig). The selected aptamer pool from SELEX round 11 was cloned and 88 individual aptamer clones were sequenced and further characterized. Seven sequence groups could be identified containing 3–8 homologous aptamer clones (Fig 1). Five sequences of the 88 individual aptamer clones were present twice in the pool (S3 Fig), but the majority of clones were orphans [30]. This indicates that the SELEX process results in a very heterogeneous aptamer pool.

**Fig 2:**
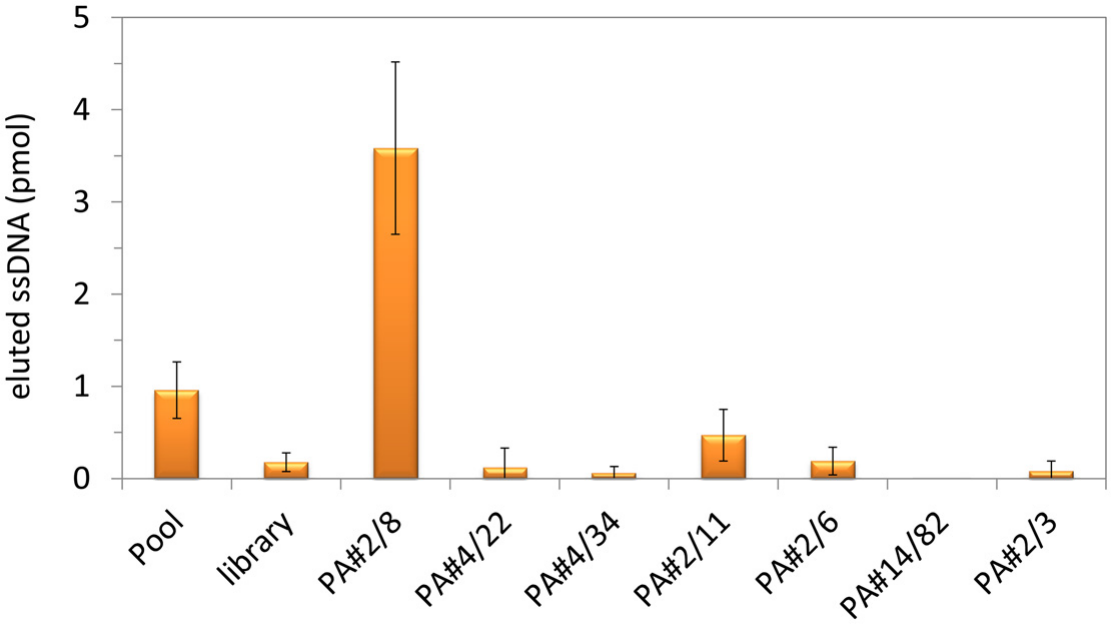
Individual binding abilities of the most abundant aptamer sequences to Protein A. The representative aptamer clones of the seven sequence groups were tested for their binding ability to Protein A in comparison to the selected aptamer pool and the unselected SELEX library. Bead-based binding assays were performed using Protein A/Strep-MB and fluorescein-labeled ssDNA. Target-bound aptamers were eluted and quantified.

The representative aptamer clones of the seven sequence groups listed in Fig 1 were screened for their individual abilities to bind to Protein A as selection target (Fig 2). According to the FluMag-SELEX conditions, the fluorescein-labeled individual aptamer clones as well as the selected aptamer pool and the starting SELEX library as negative control were incubated with Protein A/Strep-MB. After washing of the binding complexes, the bound aptamers were eluted and quantified. Unexpectedly, only aptamer PA#2/8 was able to bind to Protein A even with a much higher binding signal than the selected aptamer pool. Very small signals were observed for the other aptamer clones comparable with that of the SELEX library. These signals were in the range of the unspecific background signals of the binding assay and indicate no binding ability to Protein A. Therefore, aptamer PA#2/8 was chosen for further examinations regarding its affinity and specificity to the target and sequence optimization by truncations.

**Fig 3:**
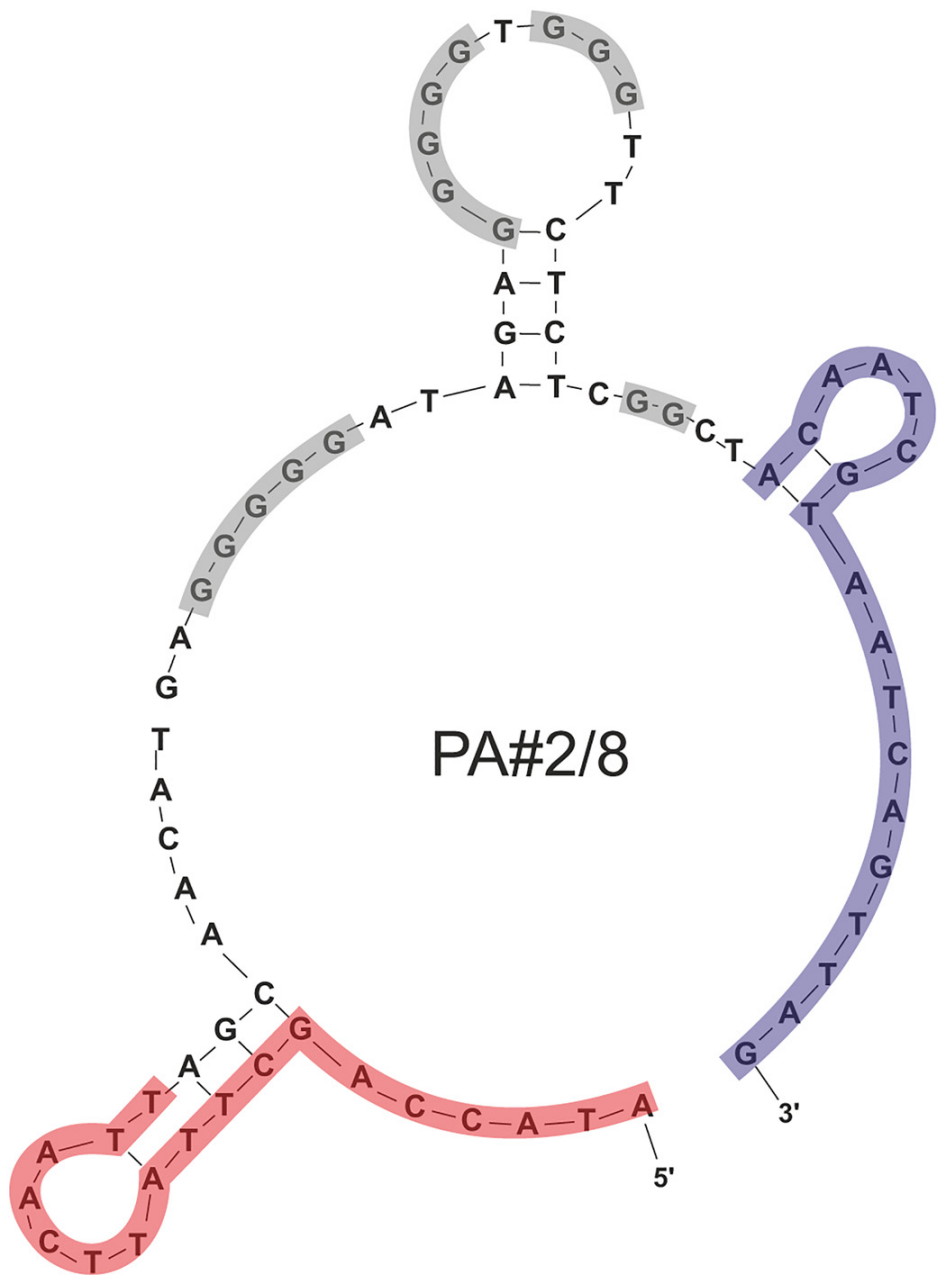
Potential secondary structure of aptamer PA#2/8. The primer binding sites (18 nt each) at the 5’- and 3’-end are highlighted in red and blue, respectively. The four G-stretches in the intern sequence region are highlighted in grey.

Aptamer PA#2/8 has a length of 76 nt. Secondary structure analysis was performed by means of the free-energy minimization algorithm using the web based tool mfold with salt correction [26–28]. According to this computational structure prediction, both primer binding sites are partly involved in a stem-loop structure with a third stem-loop formed by nucleotides of the sequence region between them (Fig 3). Furthermore, this internal sequence region of 40 nt is also characterized by four stretches of guanines with different numbers of G-residues (**GGGGG**-*5N*-**GGGGG**-*N*-**GGG**-*7N*-**GG**). Such guanine-rich sequences have the capacity to fold into so called G-quadruplex structures, but which cannot be predicted by mfold. Therefore, Fig 3 only shows potential secondary structure elements derived from the specific aptamer sequence and cannot give the final aptamer conformation. G-quadruplexes represent a special tertiary conformation of DNA and consist of two or more planar arrays of four guanines and intervening loops. They can be formed by intramolecular folding of a single stranded DNA molecule or by intermolecular association of two or four DNA molecules [31]. Biophysical methods like UV and circular dichroism (CD) spectroscopy or native gel electrophoresis typically have been applied to analyze potential G-quadruplexes. Since the beginning of the aptamer research many of the described aptamers display G-quadruplex structures and were selected mainly for proteins, like human thrombin, but also for some other targets, like ochratoxin A or ethanolamine [32–34].

**Fig 4:**
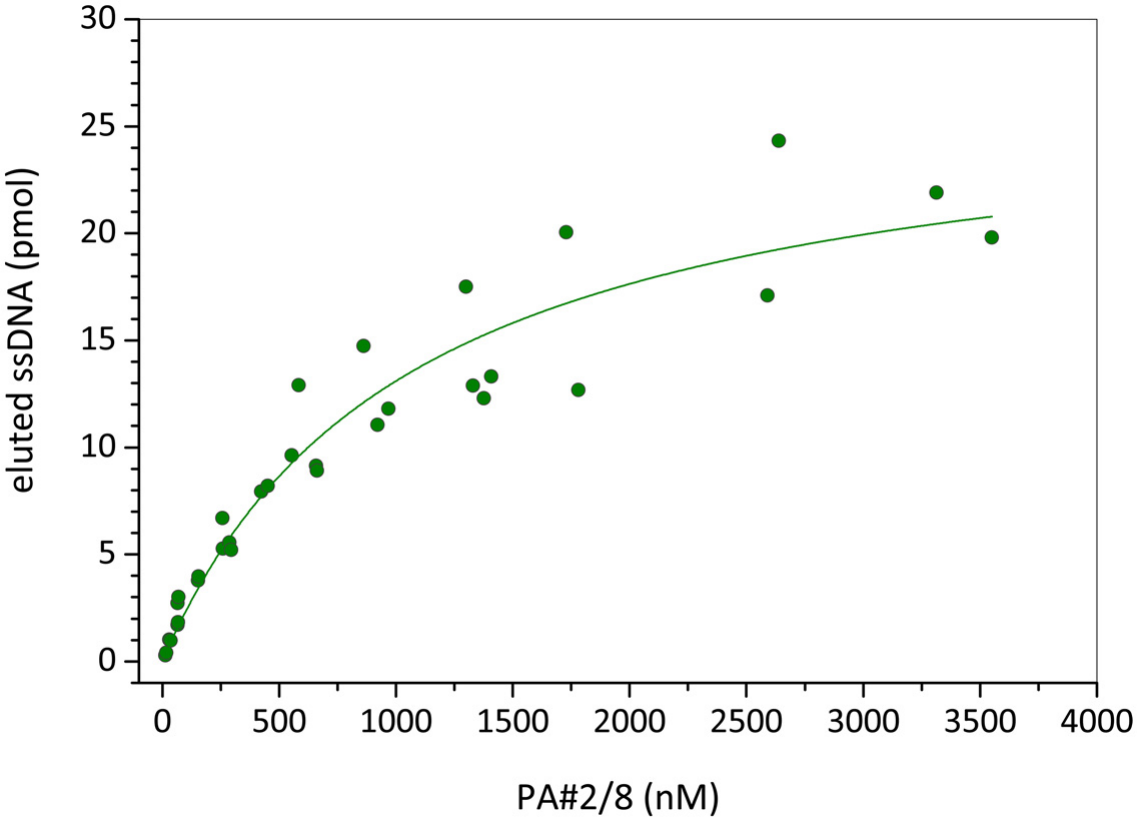
Binding curve of aptamer PA#2/8 obtained by bead-based binding assays. A constant number of Protein A/Strep-MB in each assay and a concentration series of the fluorescein-labeled aptamer were used. The dissociation constant (*K*_D_) of 1.06 ±0.2 μM was calculated by nonlinear regression analysis.

Bead-based binding assays according to the selection conditions revealed a concentration dependent binding of aptamer PA#2/8 to Protein A/Strep-MB (Fig 4). A concentration range of 12 nM—3600 nM fluorescein-labeled aptamer was applied to a series of individual binding assays with a constant number of target-modified beads. On the basis of these binding data, a saturation curve was obtained and a dissociation constant of *K*_D_ = 1.06 ±0.2 μM was calculated.

Besides the bead-based binding assays, SPR and MST measurements were also used to investigate the interaction between aptamer PA#2/8 and Protein A in more detail and to evaluate the affinity under different assay conditions (see below). The results differ in dependence of the assay setup and detection principle, but give a deeper insight in the functionality of the aptamer.

### Aptamer truncations and their effects on the binding ability to Protein A

**Fig 5:**
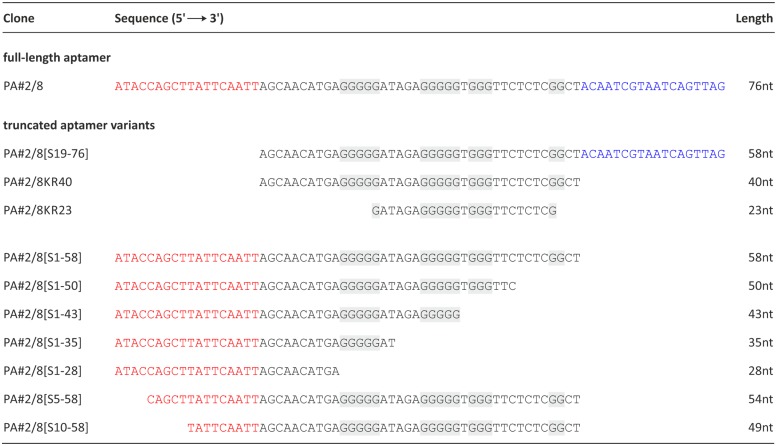
Full-length aptamer PA#2/8 and truncated aptamer variants. The primer binding sites at the 5’- and 3’-end are colored in red and blue, respectively. The G-stretches in the internal sequence region are highlighted in grey.

**Fig 6:**
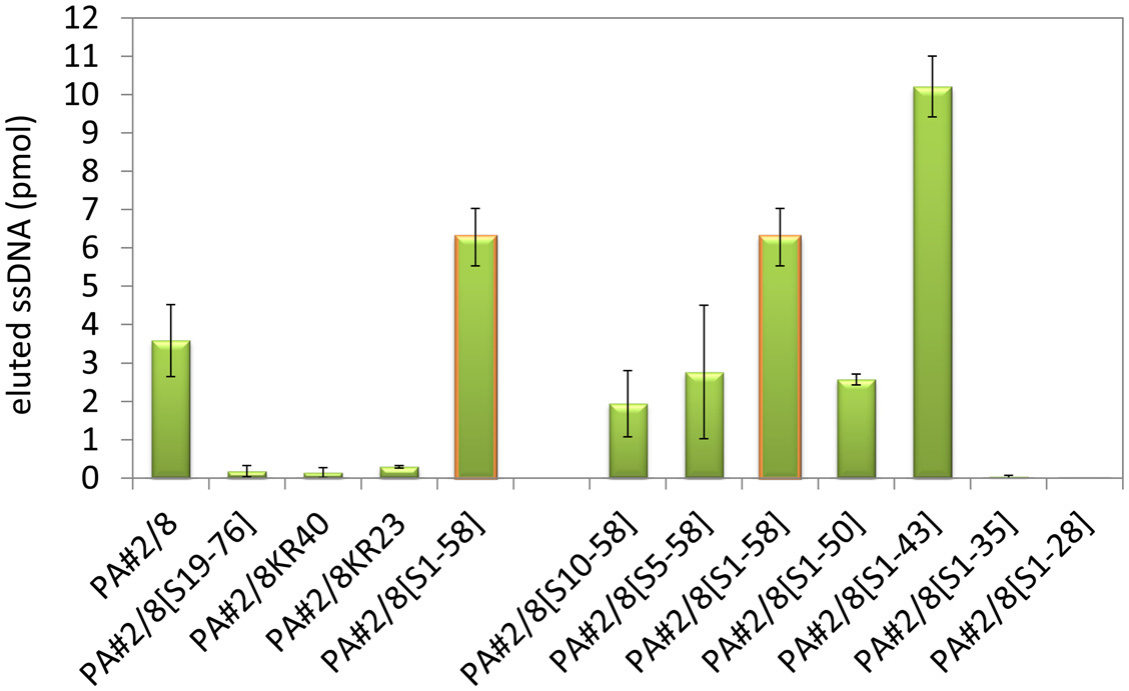
Binding abilities of the truncated aptamer variants in comparison to the full-length aptamer PA#2/8 to Protein A. Bead-based binding assays were performed using Protein A/Strep-MB and fluorescein-labeled ssDNA. Target-bound aptamers were eluted and quantified.

The aptamer PA#2/8 was stepwise truncated to narrow down the aptamer sequence region responsible for target binding. All of the truncated aptamer variants were fluorescein-labeled at the 5’-end and tested for their functionality using bead-based binding assays with Protein A/Strep-MB. Firstly either one of the specific primer binding sites at the ends of aptamer PA#2/8 (PA#2/8[S19-76], PA#2/8[S1-58]) or both of them (PA#2/8KR40, PA#2/8KR23) were removed (Fig 5). Only aptamer variant PA#2/8[S1-58] was able to continue to bind to Protein A even with a higher binding signal than the full-length aptamer (Fig 6). No binding signals were observed for the variants PA#2/8[S19-76], PA#2/8KR40 and PA#2/8KR23. These results clearly indicate that the 5’-primer binding site of PA#2/8 is very important for the functionality of the aptamer and seems to be involved in its functional folding. A loss of the 18 nt at the 5’-end of aptamer PA#2/8 leads to a completely loss of its binding ability to Protein A. In contrast, the 18 nt at the 3’-end of PA#2/8 are not necessary for target binding.

The aptamer variant PA#2/8[S1-58] was further truncated. 8–30 nt were stepwise removed from the 3’-end, whereas the 5’-end remained intact resulting in the variants PA#2/8[S1-50], PA#2/8[S1-43], PA#2/8[S1-35], and PA#2/8[S1-28] (Fig 5). These truncations concerned the intern sequence region containing the four differently sized G-clusters. In each of these variants, one more G-cluster was removed from the aptamer sequence. The truncated variants PA#2/8[S1-50] and PA#2/8[S1-43] were able to bind to Protein A, which could be shown by comparative bead-based binding assays (Fig 6). But a completely loss of function was observed for PA#2/8[S1-35] and PA#2/8[S1-28]. The binding ability of PA#2/8[S1-43] seems to be improved, because of the binding signal of this truncated aptamer variant was much higher than that of PA#2/8[S1-58] as well as of the full-length aptamer PA#2/8. A clearly reduced binding signal was found for PA#2/8[S1-50], which represents a sequence truncation between the best binding variants PA#2/8[S1-58] and PA#2/8[S1-43]. The computational secondary structure prediction by mfold (http://mfold.rna.albany.edu/?q=mfold) for PA#2/8[S1-50] (S4 Fig) exhibits an additional, more complex but energetically favorable structure, different to those of the other truncated aptamer variants, characterized by the typical stem-loop at the 5’-end (also present in the potential 2D-structure of the full-length aptamer PA#2/8, Fig 3). Such a different folding could be the reason for the reduced binding ability of PA#2/8[S1-50]. Two further aptamer variants were truncated at the 5’-end, whereas the 3’-end of PA#2/8[S1-58] remained unmodified. Aptamer variant PA#2/8[S5-58] is characterized by removing only 4 nt from the 5’-end not affecting the stem-loop structure at the 5’-region. 9 nt were removed from the 5’-end in variant PA#2/8[S10-58] resulting in disruption of the stem-loop structure (S4 Fig). The binding ability to Protein A of both aptamer variants is maintained but at strongly reduced level in comparison to PA#2/8[S1-58].

Taken together the results of the truncation experiments, the 76 nt long aptamer PA#2/8 can be shortened at the 3’-end down to 58 nt and 43 nt, respectively, even with improved binding ability to Protein A. In contrast, an intact 5’-end including the primer binding site is very important for the functionality of the aptamer.

### Immobilization orientation of the aptamer affects its functionality in SPR measurements

**Fig 7:**
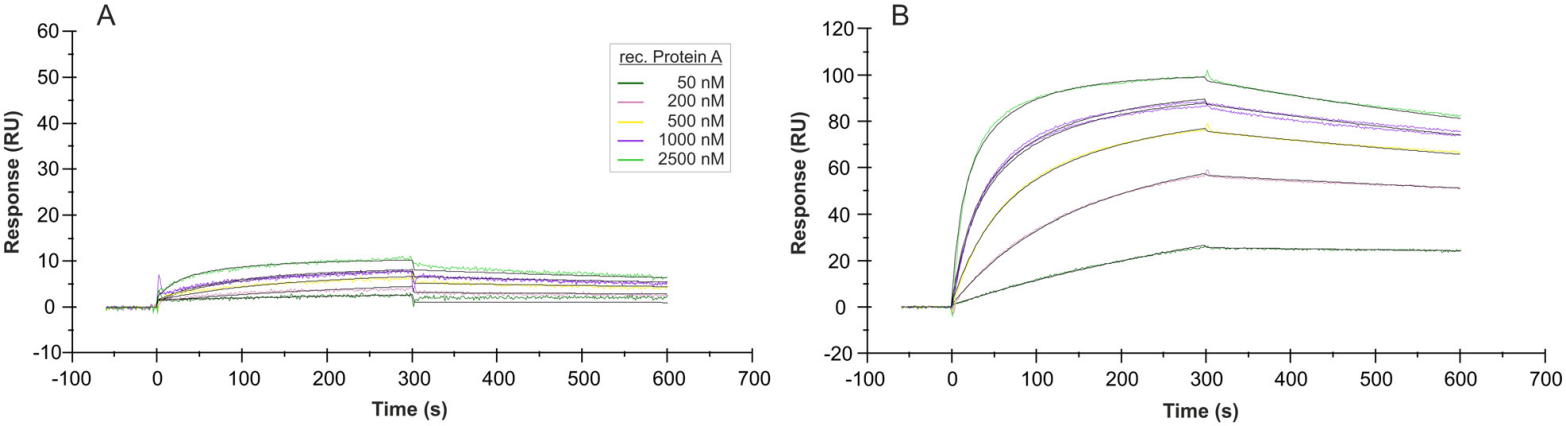
SPR interaction analyses concerning the immobilization orientation of aptamer PA#2/8. Biacore X100 / sensor chip CAP / ligand: 5’-biotinylated aptamer PA#2/8 (A) or 3'-biotinylated aptamer PA#2/8 (B) / analyte: recombinant Protein A with different concentrations (50–2500 nM, 1000 nM in duplicate). Double-referenced sensorgrams are shown (reference surface modified with unselected SELEX library, buffer injection). Black lines represent the fit to bivalent analyte binding model.

Surface plasmon resonance (SPR) based technologies stand for label-free detection methods and allow very efficient interaction analyses between aptamers and their targets on a surface. In this work, the Biacore X100 instrument was used to characterize the binding features of aptamer PA#2/8 to Protein A. In most of the experiments, end-labeled aptamer with biotin was immobilized on the streptavidin-modified sensor surface of sensor chip CAP of the Biotin CAPture Kit, and Protein A was injected as analyte across this surface for interaction with the aptamer. The first experiments have clearly shown that in case of aptamer PA#2/8 the immobilization site of this aptamer is very important for the maintenance of its functionality to bind Protein A. Immobilization of aptamer PA#2/8 at its 5’-end resulted in a very weak binding of Protein A to the aptamer layer on the sensor surface (Fig 7). In contrast, a very good and concentration-dependent binding of Protein A could be observed using a sensor surface with 3’-immobilized aptamer. This indicates that a free 5'- end of the aptamer is necessary for its correct folding as basis for formation of the binding complex with the target. These findings are in accordance with the results of the truncation experiments showing that an intact 5’-primer binding site of the aptamer must be involved in the functional folding for binding to Protein A.

### SPR measurements with regard to affinity and specificity of the aptamer

**Fig 8:**
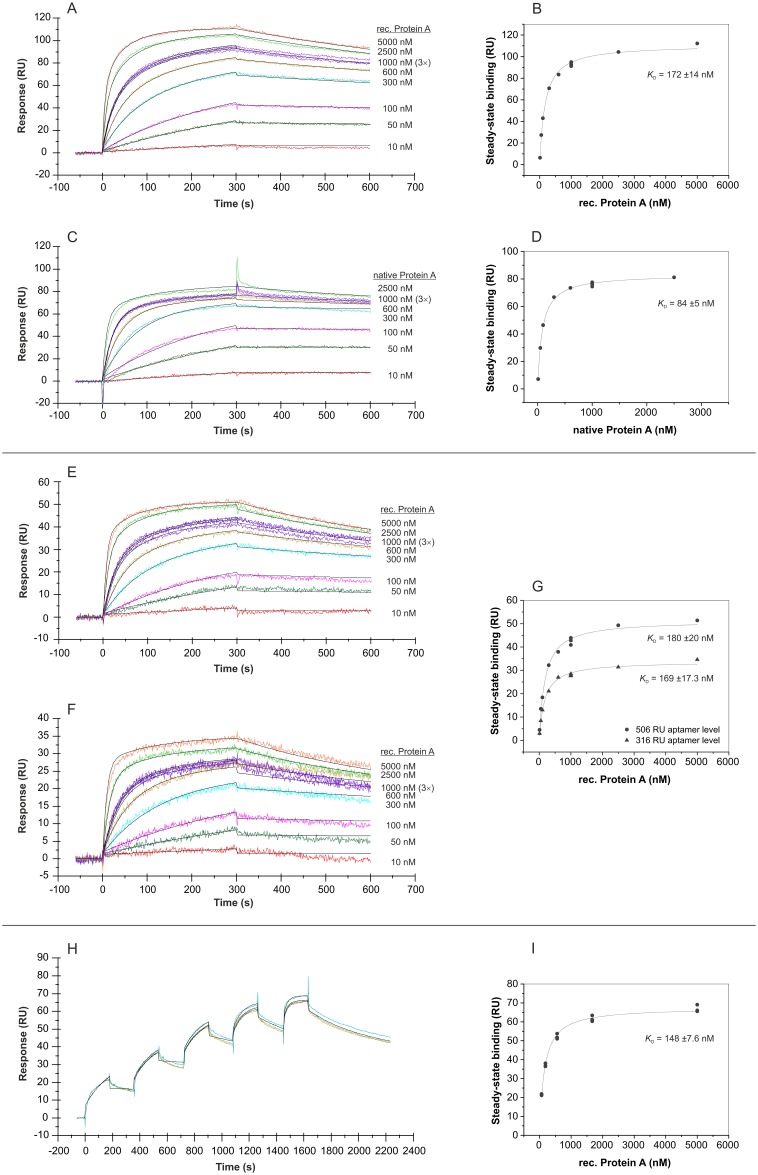
SPR interaction analyses regarding the affinity of aptamer PA#2/8. Biacore X100 / sensor chip CAP / ligand: 3'-biotinylated aptamer PA#2/8 with immobilization levels of 1086 RU (A), 1158 RU (C), 506 RU (E), 316 RU (F) / analyte: recombinant and native Protein A with different concentrations (10–5000 nM, 1000 nM in triplicate) / single-cycle mode (H) with an aptamer level of 516 RU and sequential injections of five ascending concentrations of recombinant Protein A (62, 185, 556, 1667, 5000 nM) as triplicate. Double-referenced sensorgrams are shown (reference surface modified with unselected SELEX library, buffer injection). Black lines represent the fit to bivalent analyte binding model. The corresponding plots (B, D, G, I) of steady-state binding from the end of the association phases against analyte concentration were used to calculate the steady-state affinity.

In addition to the bead-based binding assays, SPR measurements were used to analyze the binding affinity of aptamer PA#2/8 to its target Protein A in more detail. In the first experimental setup, the sensor surface was build up by immobilization of 3’-biotinylated aptamer on the streptavidin-coated sensor chip, and a concentration series of Protein A (native or recombinant) in the range of 10–5000 nM was injected. An aptamer level of 1000–1200 RU on the sensor surface was adjusted in each experiment over the injection time during the immobilization of the aptamer. The sensorgrams in Fig 8A and 8C reveal a very tightly and stable binding behavior of both recombinant and native Protein A to the immobilized aptamer PA#2/8. Fig 8B and 8D show the corresponding saturation curves derived from the binding data at the end of the binding phases. Based on this, dissociation constants in the low nanomolar range were calculated: *K*_D_ = 172 ±14 nM for the recombinant Protein A and *K*_D_ = 84 ±5 nM for the native Protein A.

The sensorgrams in Fig 8 also show the fit of the binding data to a bivalent analyte binding model as overlay. A simple 1:1 interaction model did not fit the data very well. Protein A is widely known for its ability to bind immunoglobulins from different species, especially IgG. It is composed of five highly homologous Ig-binding domains. Such specific binding sites of proteins are often preferred interaction sites for aptamers, when using these proteins as aptamer selection target [35]. In case of aptamer PA#2/8, it could therefore interact with Protein A at the same multiple sites as immunoglobulins. The binding curves in Fig 8 reflect this hypothesis. This means that one molecule Protein A could bind at least to two aptamers at the sensor surface, which gives stabilization on the ligand-analyte complex without increasing the response signal but with affecting the equilibrium constant. Such effect caused by multivalent binding analytes is also called avidity effect. Avidity is well known for antibody-antigen interactions because of the bivalent nature of the antibodies, which are able to bind to two antigen molecules simultaneously. Avidity effects will slow down the dissociation rate yielding enhanced affinity values compared to those measured from a 1:1 interaction. Reducing the density of the monovalent ligand on the sensor surface or immobilization of the multivalent binding partner can avoid the avidity effects. Experiments with a reduced aptamer density on the sensor surface down to a level of 506 RU or 316 RU still resulted in a comparable binding behavior of Protein A with dissociation constants of *K*_D_ = 180 ±20 nM and *K*_D_ = 169 ±17.3 nM, respectively (Fig 8E–8G).

The work of Cheung et al. gives an example of a more complex mechanism of aptamer-target interaction [36]. They selected a new DNA aptamer for the target *Plasmodium* lactate dehydrogenase (PfLDH). Using X-ray crystallography, they observed a binding mechanism between aptamer and target, which is characterized by the bivalent nature of the tetrameric protein PfLDH combined with the heterogeneity of each interaction site. This means that two aptamers can bind per protein and in addition, the aptamer comprise two sequence regions (internal loop, apical tetra-loop) for a distinct interaction with the protein at each binding interface. This was also reflected by SPR measurements with aptamer and immobilized protein applying a heterogeneous ligand model for fitting the sensorgrams. This work provides a deeper understanding of the potential molecular binding mechanisms between an aptamer and its target.

Fig 8H and 8I show an example of a single-cycle experiment in contrast to commonly used multi-cycle experiments. The single-cycle mode reduces assay times as well as the number of chip regenerations required. Increasing Protein A concentrations were sequentially injected to the aptamer-modified sensor surface within one cycle. A dissociation constant of *K*_D_ = 148 ±7.6 nM was calculated based on these binding data.

**Fig 9:**
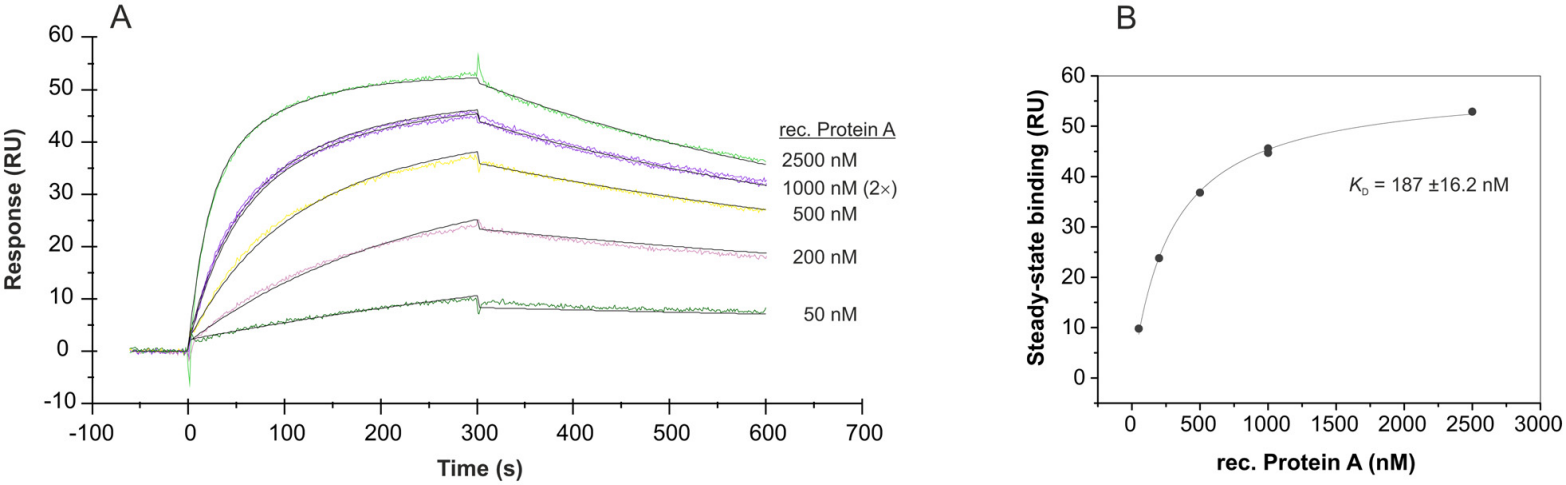
SPR interaction analysis regarding the affinity of the truncated aptamer PA#2/8[S1-58]. Biacore X100 / sensor chip CAP / ligand: 3'-biotinylated truncated aptamer PA#2/8[S1-58] with immobilization level of 1030 RU / analyte: recombinant Protein A with different concentrations (50–2500 nM, 1000 nM in duplicate). Double-referenced sensorgram (A) is shown (reference surface modified with unselected SELEX library, buffer injection). Black lines represent the fit to bivalent analyte binding model. The corresponding plot (B) of steady-state binding from the end of the association phases against analyte concentration is used to calculate the steady-state affinity.

In addition to the full-length aptamer, the 3’-biotinylated truncated variant PA#2/8[S1-58] was also used as ligand for SPR measurements (Fig 9). The binding behavior of Protein A was similar as observed to the full-length aptamer, but the sequence truncation seems to result in a slightly shift of the dissociation constants to *K*_D_ = 287 ±16.2 nM.

**Fig 10:**
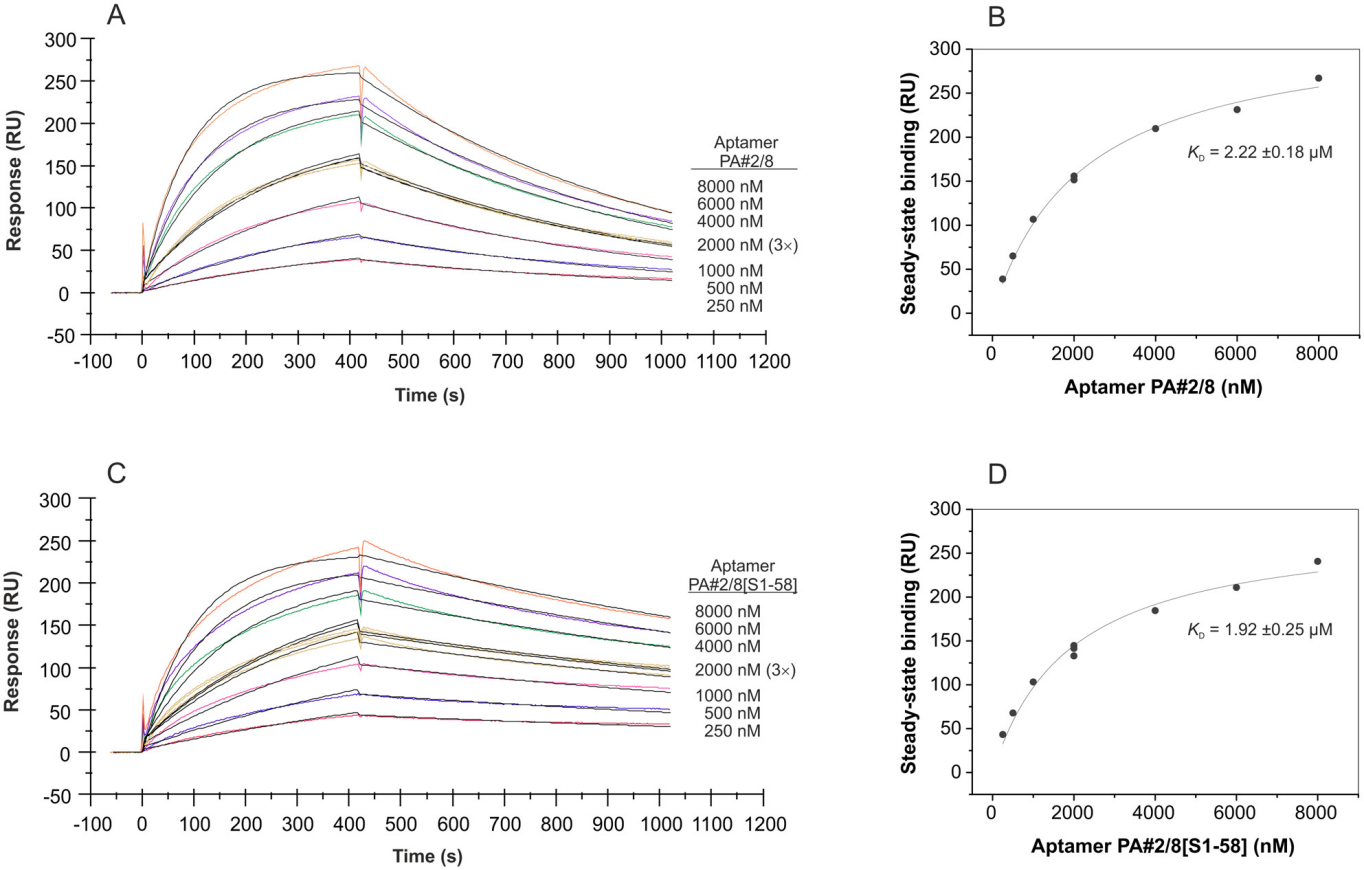
SPR interaction analyses applying the aptamers PA#2/8 and PA#2/8[S1-58] as analyte. Biacore X100 / sensor chip CAP / ligand: biotinylated native Protein A with immobilization level of ~600 RU / analyte: 5’-fluorescein-labeled aptamer PA#2/8 (A) or truncated variant PA#2/8[S1-58] (C) with different concentrations (250–8000 nM, 2000 nM in triplicate). Double-referenced sensorgrams are shown (blank reference surface without Protein A, buffer injection). Black lines represent the fit to 1:1 binding model. The corresponding plots (B, D) of steady-state binding from the end of the association phases against analyte concentration are used to calculate the steady-state affinity.

In the second and reverse experimental setup, the sensor surface was build up by immobilization of biotinylated native Protein A on the streptavidin-coated sensor chip, and a concentration series of aptamer in the range of 250–8000 nM was injected. A Protein A level of ~600 RU on the sensor surface was used in each experiment. The binding curves presented in Fig 10A and 10C clearly differ from those obtained with the aptamer immobilized on the sensor surface. Using the aptamer PA#2/8 as analyte, the binding curves are characterized by a slower association and a faster dissociation of the binding complexes. Transforming the binding data into a saturation curve (Fig 10B), the dissociation constant was calculated to be in the micromolar range with *K*_D_ = 2.22 ±0.18 μM. This decrease of the affinity was expected following the hypothesis that Protein A comprises more than one binding site for the aptamer. The supposed multivalent nature of Protein A would not appear in this sensorgram applying the reverse experimental setup. A similar dissociation constant of *K*_D_ = 1.92 ±0.25 μM was calculated for the truncated aptamer variant PA#2/8[S1-58] (Fig 10D). The data were fitted in this case to the 1:1 interaction model yielding the following kinetic parameters for the full-length aptamer PA#2/8: *k*a = 1.23 × 10^3^ M^-1^s^-1^, *k*d = 1.66 × 10^−3^ s^-1^, *K*_D_ = 1.35 μM and for the truncated aptamer PA#2/8[S1-58]: *k*a = 1.21 × 10^3^ M^-1^s^-1^, *k*d = 6.32 × 10^-4^ s^-1^, *K*_D_ = 0.522 μM. The binding of the truncated aptamer variant to the Protein A-modified sensor surface seems to be more stable than that of the full-length aptamer. This is represented by the dissociation phases in the sensorgrams and also by the lower dissociation rate constant. In consequence of that, a significantly lower *K*_D_ was obtained for the truncated aptamer variant. Comparing the steady-state affinities derived from the saturation curves with the affinities obtained by kinetic analysis, big differences between both calculation modes become apparent. If the steady-state of a binding is not really reached during the association phase as seen in the sensorgrams in Fig 10A and 10C, the calculation of the steady-state affinity is more difficult and can therefore differ from the results of the kinetic analysis.

**Fig 11:**
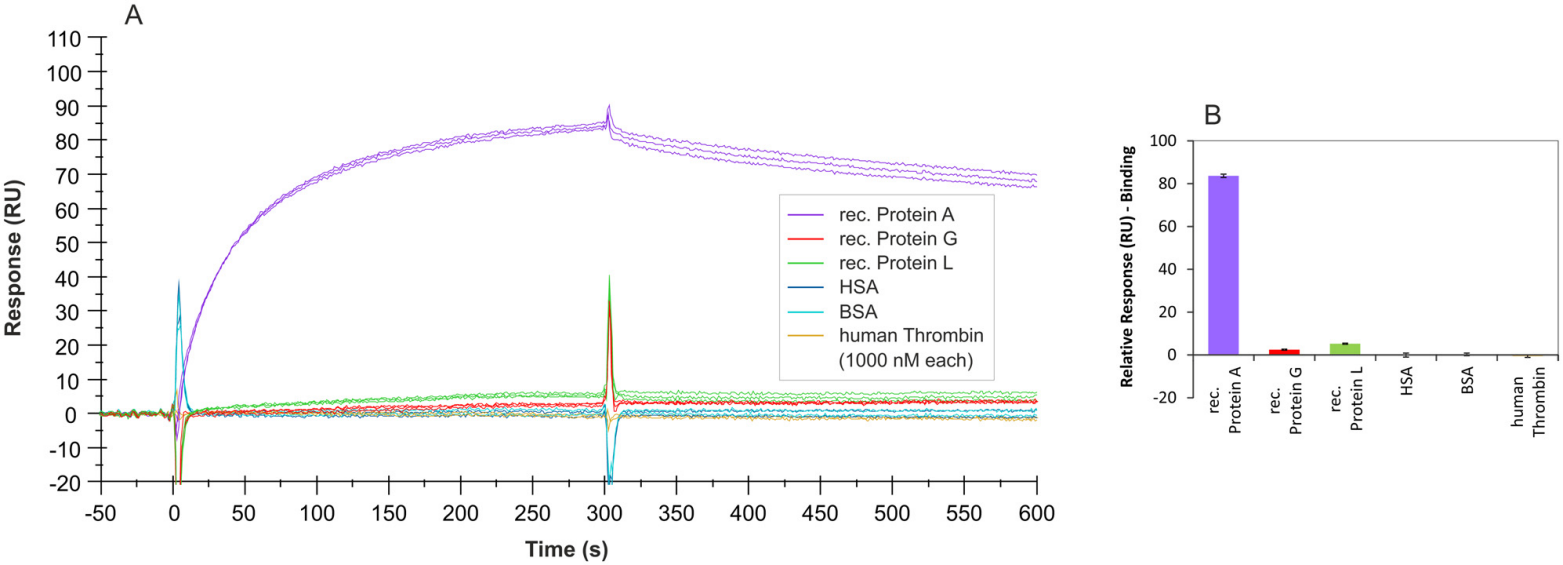
SPR interaction analyses regarding the specificity of aptamer PA#2/8. Biacore X100 / sensor chip CAP / ligand: 3'-biotinylated aptamer PA#2/8 with immobilization level of 1000–1200 RU / analyte: different proteins with a concentration of 1000 nM each (recombinant Protein A, Protein G, and Protein L in triplicate; HSA, BSA, and human Thrombin in duplicate). Double-referenced sensorgrams (A) are shown (reference surface modified with unselected SELEX library, buffer injection). Bar graph (B) of binding levels of the different proteins from the end of the association phases (after 300 s of injection) is presented.

SPR measurements were also used to characterize the binding features of aptamer PA#2/8 regarding its specificity. An overlay of sensorgrams showing interactions between different proteins and immobilized aptamer is presented in Fig 11A. Aptamer PA#2/8 effectively distinguishes the target Protein A from the functionally related proteins Protein G and Protein L. Both proteins are also immunoglobulin-binding proteins and like Protein A commonly used to purify, immobilize, or detect immunoglobulins [5]. On the other hand, there are strong differences between these three proteins concerning their origins or structural features. Furthermore, each of these proteins has a different immunoglobulin-binding profile, e.g., in terms of the binding site, the species, and type of Ig. Protein G is a bacterial cell wall protein from group G *Streptococci* [37–39]. It contains two Ig-binding domains as well as sites for albumin and cell surface binding, which were eliminated from the recombinant Protein G expressed in *E*.*coli* and used in this work. Protein L is a bacterial cell surface protein from *Peptostreptococcus magnus*. In contrast to Protein A and Protein G, which bind to the Fc region of immunoglobulins, Protein L binds Igs through light chain interactions, but without interfering with the antigen-binding site, and also binds to single chain variable fragments (scFv) and Fab fragments. The recombinant variant of this protein expressed in *E*.*coli* was used in this work [40–42].

In addition to that, no unspecific binding of aptamer PA#2/8 was found to human serum albumin (HSA), bovine serum albumin (BSA), and human thrombin (Fig 11).

### Identification of the aptamer binding site in Protein A

**Fig 12:**
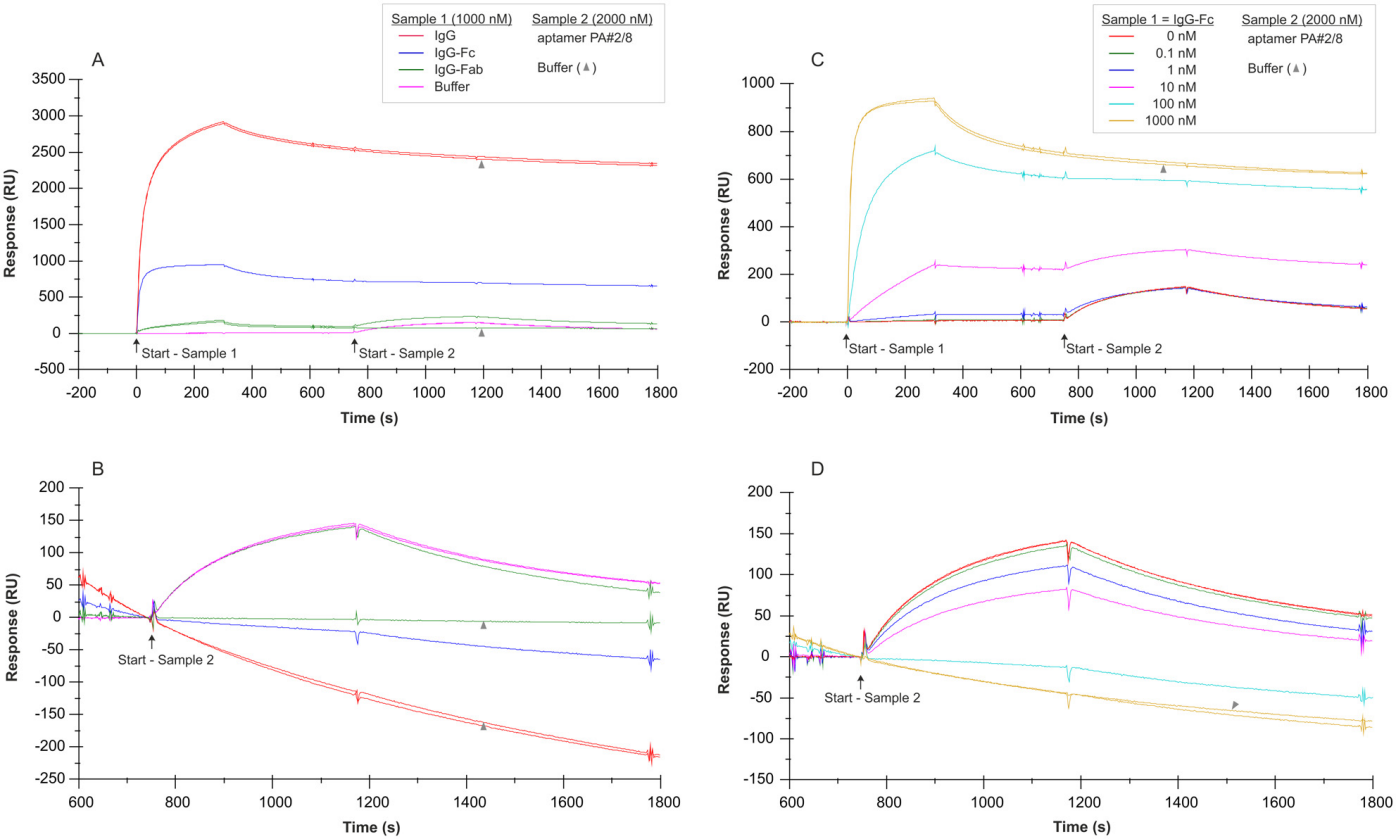
SPR interaction analyses regarding the aptamer binding site in Protein A. Biacore X100 / sensor chip CAP / ligand: biotinylated Protein A with immobilization level of ~560 RU / two-step analyte binding without regeneration in between, (A-B) analyte 1 = sample 1: human IgG, IgG-Fc fragment, IgG-Fab fragment with a concentration of 1000 nM each, or buffer, (C-D) analyte 1 = sample 1: concentration series of human IgG-Fc in the range of 0–1000 nM, (A-D) analyte 2 = sample 2: 2000 nM 5’-fluorescein-labeled aptamer PA#2/8 or buffer. Double-referenced sensorgrams are shown (blank reference surface without Protein A, buffer injection). Binding of sample 1 followed by sample 2 is shown in (A) and (C) with alignment to injection start of sample 1. In (B) and (D) only binding of sample 2 with alignment to injection start of sample 2 is shown.

Two-step analyte binding experiments without regeneration in between were performed using the Biacore X100 instrument to prove the hypothesis that aptamer PA#2/8 interacts with Protein A at the same sites as immunoglobulins. For this purpose, biotinylated Protein A was immobilized on the sensor surface (~560 RU). Different cycles were run using human IgG, IgG-Fc fragment, IgG-Fab fragment (1000 nM each), or only buffer for the first sample injection to allow binding to Protein A followed by dissociation (Fig 12A). Afterwards aptamer PA#2/8 (2000 nM) or only buffer representing the second sample was directly injected for binding. Not until after the second dissociation phase the sensor surface was regenerated for a new cycle. As expected, human IgG and its Fc fragment are able to bind very tightly and stable to Protein A on the sensor surface (Fig 12A), and resulted in a very strong signal. A relatively weak binding signal was observed for the human Fab fragment. Less common is the ability of Protein A to bind the Fab fragment of immunoglobulins, especially the Fab heavy chain V_H_3 family [7, 8, 43, 44]. It was found that both Fc and Fab fragments bind to Protein A in a noncompetitive way and that they use the same Ig-binding domains, but different epitopes inside of these domains [44]. If IgG or the IgG-Fc fragment was bound to Protein A first, a subsequent interaction of aptamer PA#2/8 with the same sensor surface could not be found (Fig 12B). A typical binding signal for the aptamer was only observed after using buffer or the Fab fragment as analyte during the first binding step. Fig 12C and 12D show a similar experiment with the injection of a concentration series of IgG-Fc in the range of 0–1000 nM during the first binding step. In this case, the aptamer PA#2/8 was able to bind to Protein A afterwards in dependence of the amount of bound IgG-Fc on the sensor surface. From the results of both experiments, it can be concluded that aptamer PA#2/8 compete with IgG or IgG-Fc for binding to Protein A and therefore use the same sites as immunoglobulins for interaction with Protein A.

### Aptamer—Protein A interaction analyses using MicroScale Thermophoresis (MST)

**Fig 13:**
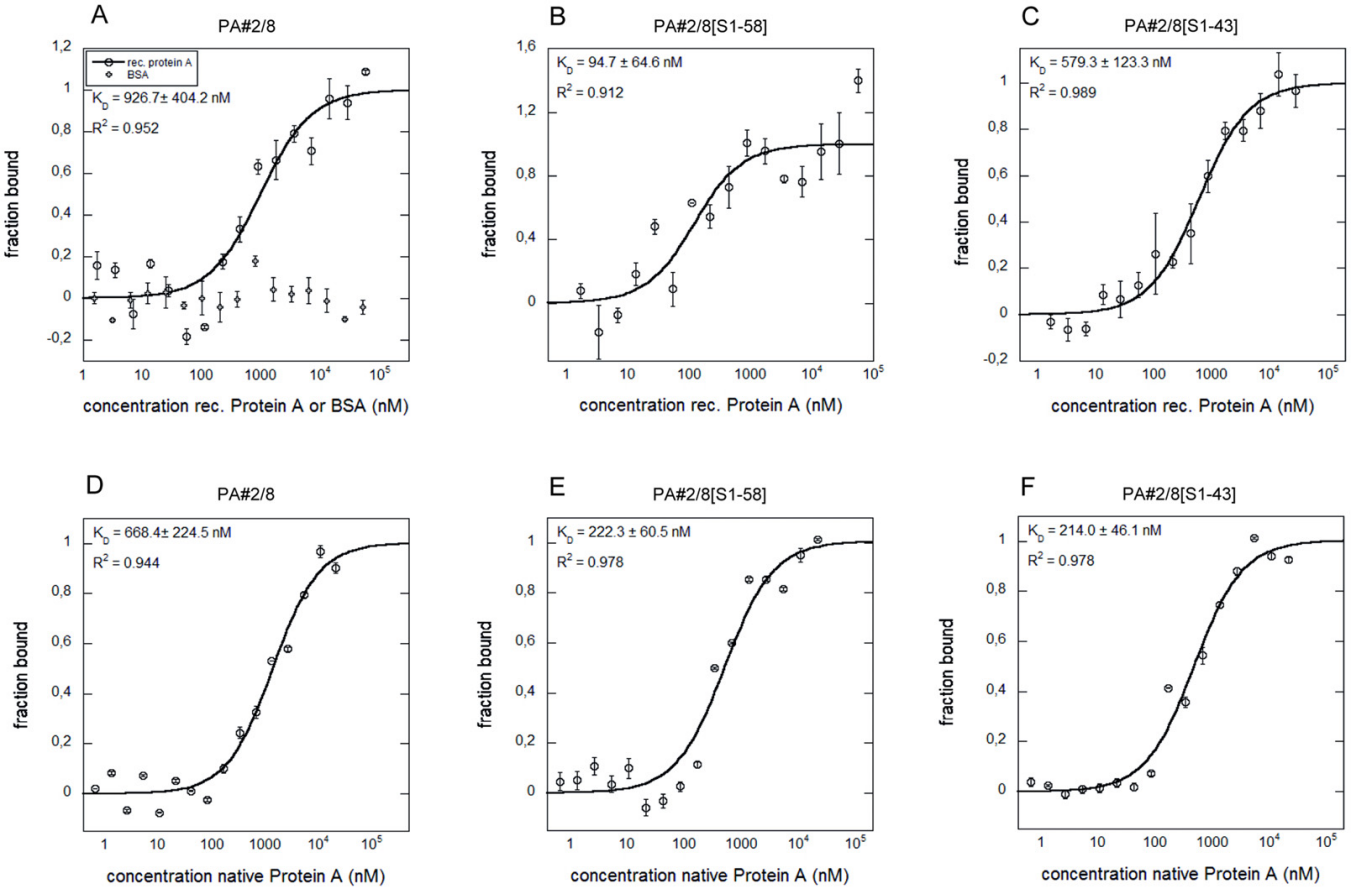
Aptamer—Protein A interactions analyzed by MST. Binding curves for the interactions of fluorescently labeled aptamer PA#2/8 (A, D) or its truncated variants PA#2/8[S1-58] (B, E) and PA#2/8[S1-43] (C, F) with recombinant or native Protein A are shown. BSA was used as negative control (A). The aptamer concentration was kept constant at 50 nM for each interaction analysis and the protein was titrated in the range from 1.69 to 55,555 nM of recombinant Protein A and from 0.33 to 10,820 nM of native Protein A, respectively. The binding data were fitted, and the dissociation constants (*K*_D_) were calculated.

**Fig 14:**
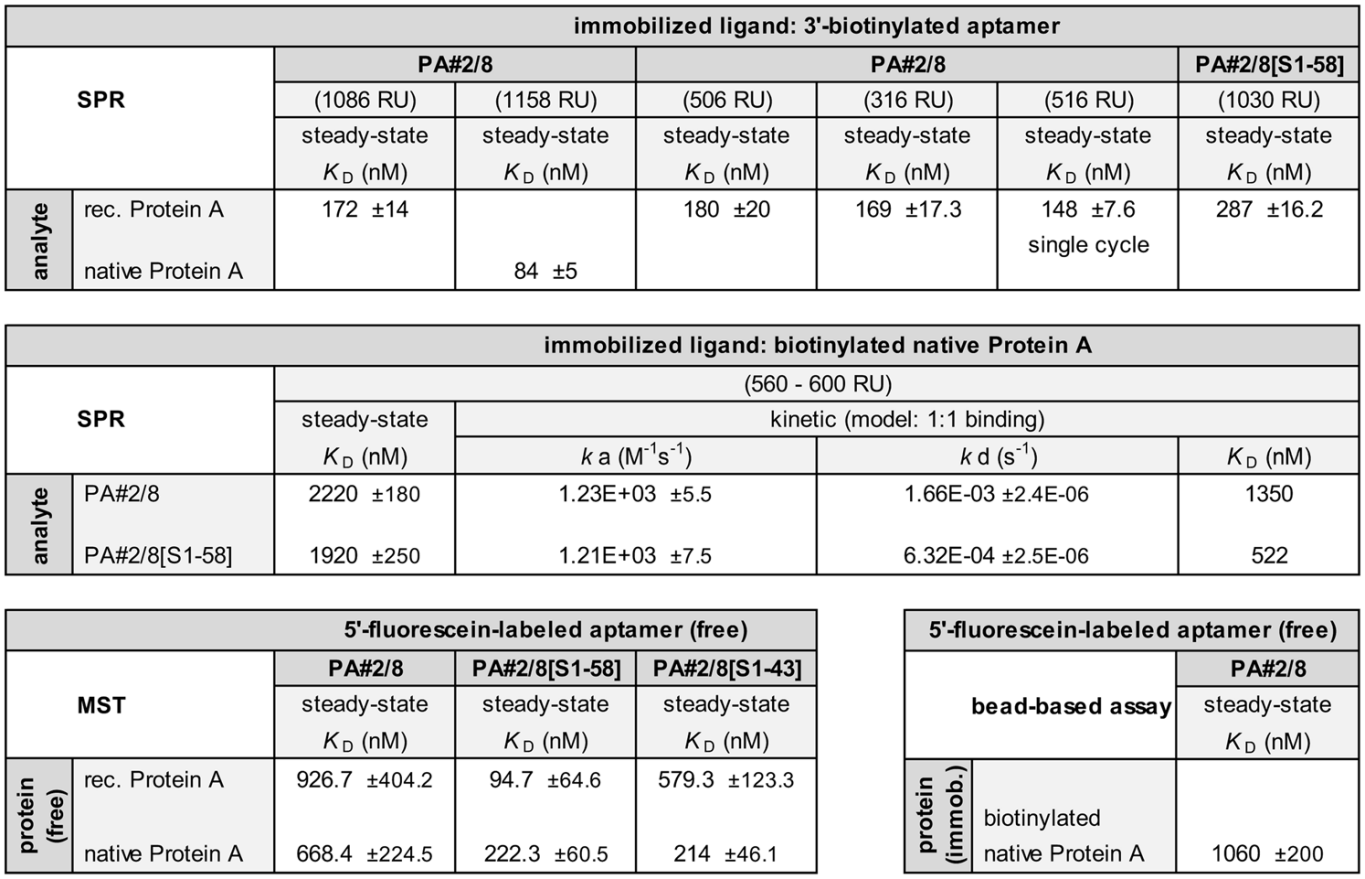
Overview of calculated dissociation constants (*K*_D_) of aptamer PA#2/8 and its truncated variants. Results of the applied assays with different measuring principles were compared.

The MicroScale Thermophoresis is a very useful technology to identify and characterize biomolecular interactions of any kind and therefore is of special interest to the field of aptamer research. It allows fast measurements in solutions without surface immobilization of one of the binding partners with free choice of buffer [45, 46]. The MST technology is based on movement of molecules in a temperature gradient. The thermophoresis of an aptamer differs significantly from the thermophoresis of an aptamer-target complex due to changes in the size, charge or hydration shell of the aptamer upon binding to the target. Such changes in the thermophoretic mobility of the aptamer are monitored using a fluorescent label. In this work, the 5’-fluorescein-labeled aptamer PA#2/8 was combined with various concentrations of Protein A (recombinant or native), and the different movement of the unbound or target-bound state of the aptamer along the temperature gradient over the assay time was measured. The resulting data were analyzed to obtain binding curves and to allow the evaluation of the binding affinities by determination of the dissociation constants (Fig 13). Avidity effects, as observed in the SPR measurements when the aptamer was immobilized on the sensor surface, should not appear using the MST experimental setup. Therefore, affinities in the low micromolar to submicromolar range were expected for the full-length aptamer and are in agreement with the results from the above sections (Fig 14). *K*_D_ values of 926.7 ±404.2 nM for the interaction with recombinant Protein A and 668.4 ±224.5 nM for the interaction with native Protein A were calculated (Fig 13A and 13D). BSA as negative control remained unbound by PA#2/8 (Fig 13A). Similar experiments were also performed with the 5’-fluorescein-labeled aptamer truncations PA#2/8[S1-58] and PA#2/8[S1-43]. Typical binding curves could be measured for both truncated variants. The 58 nt long variant PA#2/8[S1-58] even reveal a strong increase of the affinity with *K*_D_ = 222.3 ±60.5 nM for interaction with native Protein A and with a *K*_D_ = 94.7 ±64.6 nM for interaction with recombinant Protein A (Fig 13B and 13E). The affinity was the same for binding of the 43 nt long variant PA#2/8[S1-43] to native Protein A (*K*_D_ = 214 ±46.1 nM), whereas the affinity to the recombinant Protein A was determined to be lower (*K*_D_ = 579.3 ±123.3 nM) (Fig 13C and 13F). These MST results confirm differences in the binding behavior of aptamer PA#2/8 and its truncations to Protein A, which could be already concluded from the kinetic data of the SPR measurements using immobilized Protein A as ligand (Fig 14). Both truncated variants PA#2/8[S1-58] and PA#2/8[S1-43] show improved binding features to recombinant and native Protein A in comparison to the full-length aptamer.

The measurements demonstrate that the MST is a very suitable method to characterize aptamer-target interactions directly in solution without interfering effects like avidity or immobilization of one binding partner, which can alter the binding behavior [47].

Among the published aptamer developments for *Staph*. *aureus* cells, only two were focused on the specific selection of aptamers for Protein A as a cell surface component. Baumstummler et al. have selected so called SOMAmers for Protein A as selection target [21]. These SOMAmers are special DNA aptamers that contain modified nucleotides either 5-naphthylmethyl-aminocarbonyl-dU (NapdU) or 5-tryptaminocarbonyl-dU (TrpdU), which mimic amino acid side chains [48]. These features expand the chemical diversity of standard aptamers and enhance the specificity and affinity of protein-nucleic acid interactions. Dissociation constants in the subnanomolar range for the best binding SOMAmers for Protein A were measured using radiolabel affinity binding assays, and the specific recognition of intact *Staph*. *aureus* cells was shown. Friedman et al. reported the direct selection of 2’-fully modified RNA aptamers from a fGmH RNA library (2'-F-dG, 2'-OMe-dA/dC/dU) for Protein A as a model target [22]. They could demonstrate the high nuclease and serum stability of these aptamers, which were able to bind to purified Protein A with affinities in the middle to low nanomolar range and to target Protein A present on the surface of *Staph*. *aureus* cells. Beside Protein A, other specific cell surface associated structures like teichoic acid were used to select RNA aptamers targeting the gram-positive cell wall of *Staph*. *aureus* [19, 20].

A different aptamer selection strategy was described by the use of whole cells of *Staph*. *aureus* as complex selection targets, but without identifying the specific molecules to which the selected aptamers bind on the cell surface [15–18]. All of these known aptamers relating to *Staph*. *aureus* differ among each other and from PA#2/8 regarding their sequence features and thus their binding abilities.

## Conclusions

In the present work, the selection and evaluation of the DNA aptamer PA#2/8 targeting Protein A is described. The work was focused on detailed interaction analyses between aptamer and Protein A using methods with different experimental setups like bead-based binding assays, SPR measurements, and MST measurements. The functionality of the aptamer free in solution and in assays with immobilization of one of the binding partners was successfully demonstrated. The affinity of aptamer PA#2/8 to its target Protein A is strongly depending on the assay conditions and was generally found to be in the low micromolar to submicromolar range. But the affinity was increased to the low nanomolar range if avidity can play a role under the applied assay conditions. Avidity effects are possible, because Protein A is a multivalent target for the aptamer. This means that Protein A provides more than one binding site for the aptamer, which seems to be overlapping with the known binding sites of immunoglobulins.

Looking at the aptamer sequence and structural features, a stem-loop-structure at the 5’-end involving the 5’-primer binding site is essential for the functionality of the aptamer PA#2/8 and may not be removed. In contrast, truncations at the 3’-end (18–33 nt) are possible with maintenance of the functionality. PA#2/8[S1-58] was the truncated aptamer variant with the best binding features. It exhibited an improved affinity to Protein A compared to the full-length aptamer, especially observed in MST measurements. Future work will be aimed at a deeper insight in structural features. The presence of four G-stretches in the aptamer sequence implies the capacity to form a G-quadruplex structure. However, the aptamer remains functional after removing of two G-stretches as shown by the truncation experiments. Therefore, an intermolecular G-quadruplex structure composed of at least two aptamer molecules would be more likely as an intramolecular G-quadruplex. E.g., circular dichroism spectroscopy is a very beneficial way to investigate the nature of the potential G-quadruplex structure and should be the next step.

Aptamer PA#2/8 is able to specifically bind to both native and recombinant Protein A with a slightly better affinity to the native protein. A biotinylated variant of native Protein A was used as aptamer selection target in this work. Other immunoglobulin-binding proteins like Protein G and L are not recognized. Cross reactivity to BSA, HSA or thrombin could not be observed.

These described binding features of aptamer PA#2/8 make it suitable for analytical applications. Next steps should focus on the development of optimized binding assays for sensitive detection of Protein A. Protein A is known as cell surface protein and important virulence factor and therefore, serves as marker for the presence of pathogenic *Staph*. *aureus*. E.g., designing a dimeric aptamer complex would take advantage of the multivalent nature of Protein A and therefore, could improve the affinity compared to the monomeric aptamer and the stability of the aptamer-target complex. Such enhancement of binding features of aptamer-based multimeric ligands was already demonstrated [49–51]. Future studies will also focus on interaction of the aptamer with Protein A in the whole bacterial cells context. The ability of the aptamer to bind *Staph*. *aureus* cells would demonstrate its utility as detecting agent not only for Protein A but also for the corresponding bacterial pathogen.

## Supporting Information

S1 Fig FluMag-SELEX. Schematic representation of the FluMag-SELEX procedure for the selection of DNA aptamers.
(TIF)

S2 Fig Aptamer selection progress. Monitoring of the amounts of oligonucleotides eluted from target-modified magnetic beads (Protein A/Strep-MB) in each selection round. In rounds 3 and 7–11, a negative selection step (*) was introduced to remove nonspecific binding oligonucleotides, e.g., to the bead matrix (Strep-MB). No significant portion of nonspecific binding oligonucleotides was observed during the negative selection step in the indicated SELEX rounds.
(TIF)

S3 Fig Aptamer sequences, which were present twice in the selected aptamer pool. One representative per group of 5 aptamer groups with 2 homologous sequences is shown (in addition to Fig 1 of the main text listing the most abundant aptamer sequences). The specific primer binding sites at the 5’- and 3’-end of the aptamer clones are colored in red and blue, respectively.
(TIF)

S4 Fig Computational secondary structure predictions of several truncated variants of aptamer PA#2/8. The primer binding sites at the 5’-end are highlighted in red. The G-stretches in the internal sequence region are highlighted in grey.
(TIF)

S1 File DNA Aptamer Selection by FluMag-SELEX. The selection of DNA aptamers for Protein A using the FluMag-SELEX procedure is described in detail.
(PDF)
