# Supplementary figures and images for: In vitro Selection and Interaction Studies of a DNA Aptamer Targeting Protein A
Source: PLoS One. 2015 Jul 29;10(7):e0134403. doi: 10.1371/journal.pone.0134403 (PMC4519192; doi:10.1371/journal.pone.0134403)

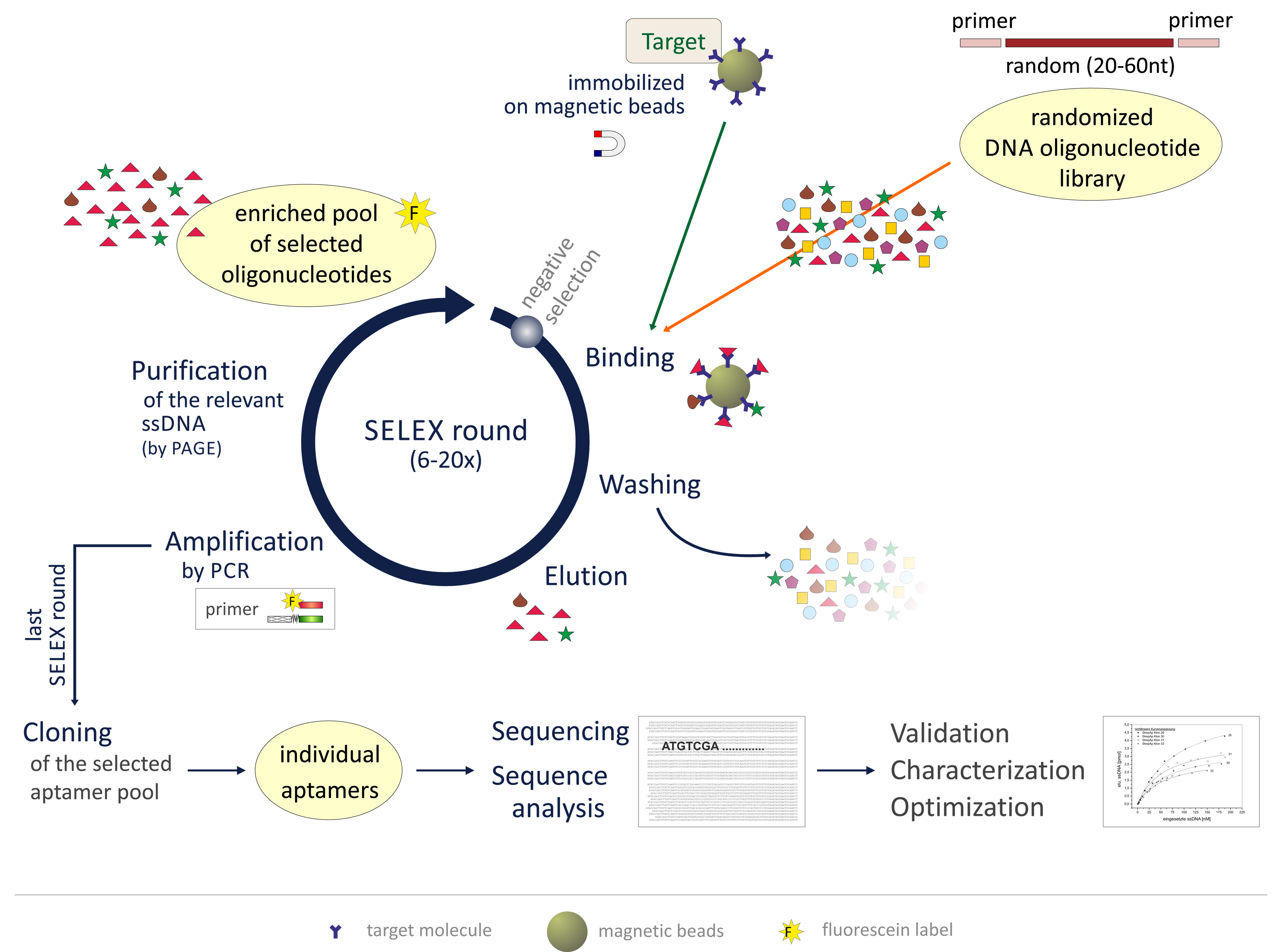

Supplement: S1 Fig — Schematic representation of the FluMag-SELEX procedure for the selection of DNA aptamers. (TIF) [file pone.0134403.s001.tif]

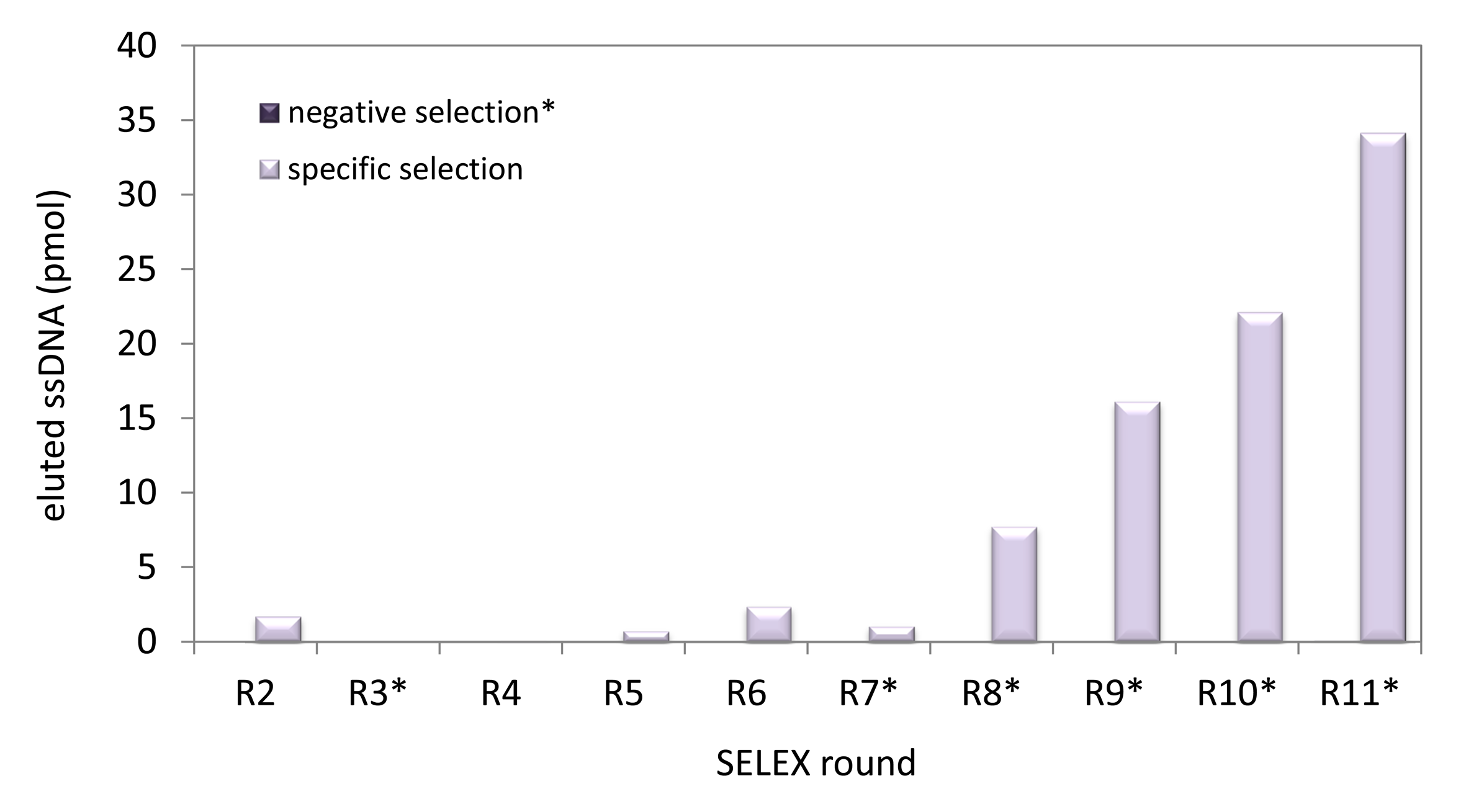

Supplement: S2 Fig — Monitoring of the amounts of oligonucleotides eluted from target-modified magnetic beads (Protein A/Strep-MB) in each selection round. In rounds 3 and 7–11, a negative selection step (*) was introduced to remove nonspecific binding oligonucleotides, e.g., to the bead matrix (Strep-MB). No significant portion of nonspecific binding oligonucleotides was observed during the negative selection step in the indicated SELEX rounds. (TIF) [file pone.0134403.s002.tif]

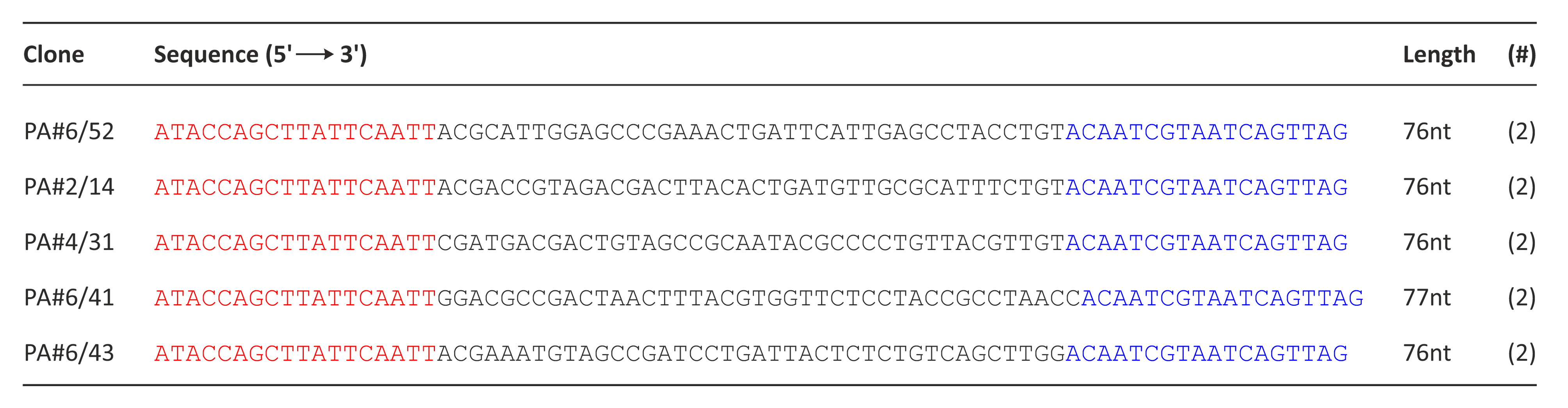

Supplement: S3 Fig — One representative per group of 5 aptamer groups with 2 homologous sequences is shown (in addition to Fig 1 of the main text listing the most abundant aptamer sequences). The specific primer binding sites at the 5’- and 3’-end of the aptamer clones are colored in red and blue, respectively. (TIF) [file pone.0134403.s003.tif]

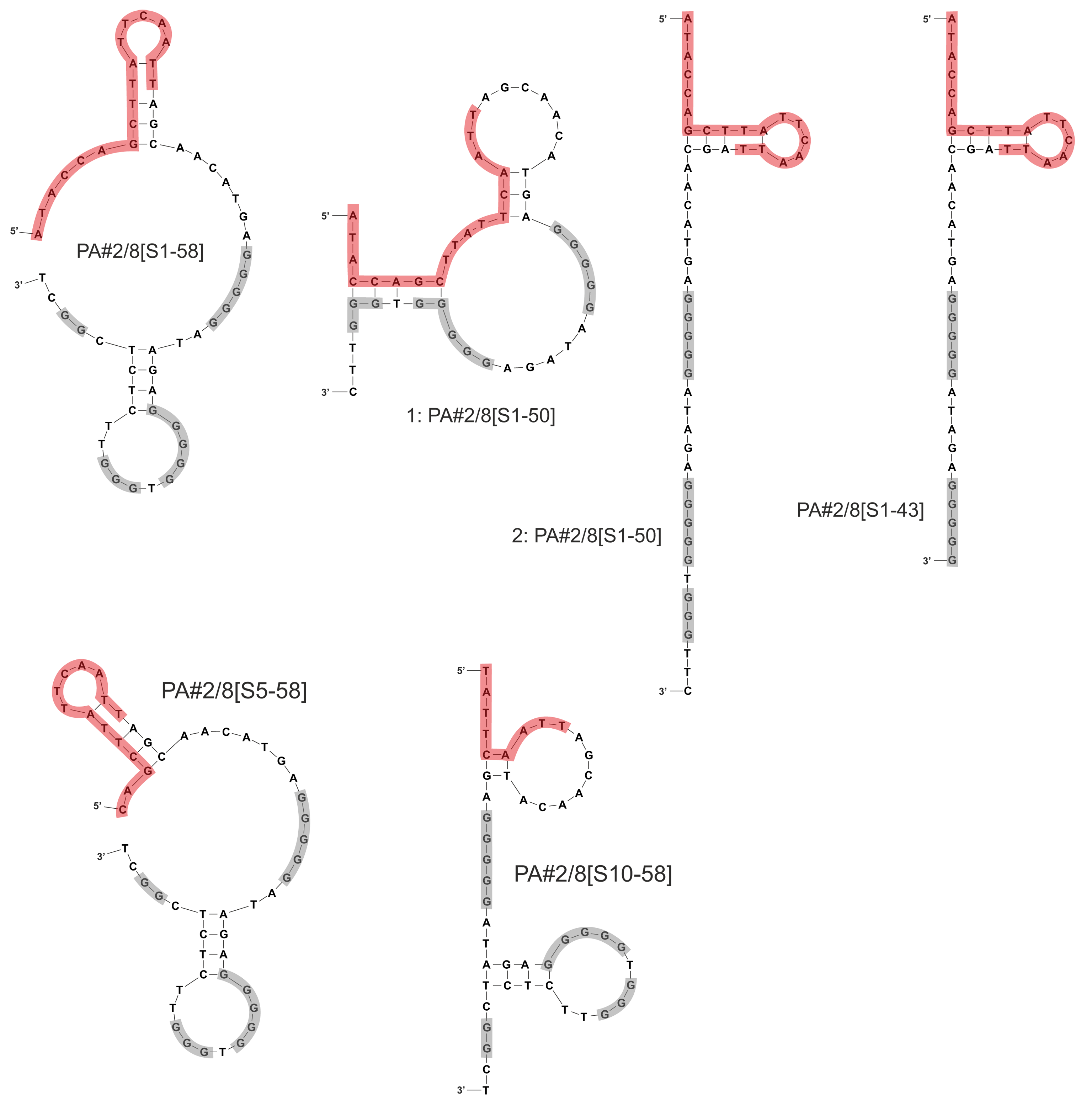

Supplement: S4 Fig — The primer binding sites at the 5’-end are highlighted in red. The G-stretches in the internal sequence region are highlighted in grey. (TIF) [file pone.0134403.s004.tif]
