# Supplementary material for: In vitro Selection and Interaction Studies of a DNA Aptamer Targeting Protein A
Source: PLoS One. 2015 Jul 29;10(7):e0134403. doi: 10.1371/journal.pone.0134403 (PMC4519192; doi:10.1371/journal.pone.0134403)
Supplement: S1 File — The selection of DNA aptamers for Protein A using the FluMag-SELEX procedure is described in detail. (PDF) [file pone.0134403.s005.pdf]

DNA aptamer selection by FluMag-SELEX

The selection of DNA aptamers for Protein A from *Staphylococcus aureus* was based on the FluMag-SELEX procedure [1]. In each SELEX round a fresh aliquot of 1×10^8 streptavidin-coated magnetic beads (Strep-MB) modified with biotinylated native Protein A (Protein A/Strep-MB) was washed three times with 500 µL selection buffer (100 mM NaCl, 20 mM Tris-HCl pH 7.6, 10 mM $MgCl_2$, 5 mM KCl, 1 mM $CaCl_2$). During the second washing step the beads, were incubated at 21°C for 5 min in selection buffer with mild shaking before magnetic separation of the beads. In parallel, the oligonucleotide pool was heated to 90°C for 8 min, immediately cooled, and kept at 4°C for 10 min followed by a short incubation at room temperature. In the initial selection round, the washed Protein A/Strep-MB were resuspended in 250 µL selection buffer containing 2.5 nmol of the pretreated SELEX library BANK-C. After an incubation of this mixture at 21°C for 30 min with mild shaking, the unbound oligonucleotides were removed by three washing steps with 250 µL selection buffer. Subsequently, the bound oligonucleotides were eluted by incubating the binding complexes in 250 µL selection buffer at 95°C for 10 min with mild shaking. This elution step was repeated once more with 250 µL selection buffer and finally with 250 µL 50 mM sodium carbonate/bicarbonate buffer, pH 9.5. All eluted oligonucleotides were pooled and amplified in 15 parallel PCR reactions. Additionally, the remaining bead bound oligonucleotides were also amplified. Each PCR reaction contained 1 µM of primers AP60 and TER-AP30, 0.2 mM dNTPs each, 1.9 mM $MgCl_2$, and 5 U HOTFire polymerase in PCR reaction buffer (80 mM Tris-HCl pH 9.5, 20 mM $(NH_4)_2SO_4$, 0.02% Tween 20) in a volume of 100 µL. Amplification conditions were 15 min at 95°C and 30 cycles of 1 min at 95°C, 1 min at 56°C, and 1 min at 72°C, and a final step of 10 min at 72°C after the last cycle. As a result, dsDNA products were obtained with a fluorescein modification at the 5'-end of the relevant sense strand and a poly-dA_{20} extension at the 5'-end of the anti-sense strand. Electrophoresis on 2.5% agarose gel was used to monitor the successful amplification and the correct size of the amplified DNA. All PCR products were pooled,

precipitated with ethanol in presence of linear polyacrylamide [2], and resuspended in 100-125 µL TE buffer (10 mM Tris-HCl pH 7.4, 1 mM EDTA). The two strands of the dsDNA differ in length due to the poly-dA_{20} extension of the anti-sense strand [3]. This was utilized for the separation of the two DNA strands in a preparative denaturing PAGE with an 8% polyacrylamide gel containing 7 M urea and 20% formamide in TBE buffer (90 mM Tris-HCl, 90 mM boric acid, 2 mM EDTA). The fluorescein-labeled sense strands could be identified in the gel by using a UV transilluminator. The corresponding DNA bands were cut out and the single stranded DNA (ssDNA) was eluted from the gel with 2 mM EDTA, 300 mM sodium acetate, pH 7.8 at 80°C for 150 min with mild shaking. After removing of the gel residues by filtering through silanized glass wool, the eluted ssDNA was precipitated with ethanol in presence of linear polyacrylamide [2] and resuspended in selection buffer. This new pool of selected and fluorescein-labeled oligonucleotides was used for the next round of selection.

The fluorescein label attached to the oligonucleotides from round two onwards enables the quantification of the oligonucleotides present in the SELEX fractions like oligonucleotide solution before and after the binding reaction to the target, washing solutions, and especially the target bound fraction of oligonucleotides after heat elution. This is important in order to assess the aptamer selection progress over several SELEX rounds.

The selection procedure in round 1 - 6 was identical, whereas from round 7 some modifications concerning the target bound oligonucleotides were made (see above). Only those oligonucleotides, which were eluted twice in 250 µL selection buffer, were subsequently pooled, amplified, and processed for the next SELEX round. To remove nonspecific binding oligonucleotides, a negative selection step was introduced in SELEX round 3 and in rounds 7 - 11. The oligonucleotide pool was exposed to unmodified Strep-MB prior to positive selection with Protein A/Strep-MB in these rounds. After washing, the oligonucleotides bound to the Strep-MB were eluted by heat treatment as described above and also quantified by fluorescence detection.

As the SELEX rounds progressed and an enrichment of target bound oligonucleotides could be observed, the stringency was increased by increasing the number, length, and volume of washing steps of the binding complexes (round 9 - 11: six washing steps with 250 µL selection buffer; additional in round 10 - 11: incubation of the binding complexes at 21°C for 15 min with mild shaking during the fourth washing step and increasing the volume to 500 µL for the fifth and sixth washing step).

The selected aptamer pool from SELEX round 11 was amplified with the unmodified primers AP10 and AP30 and subsequently cloned into the vector pCR2.1-TOPO (TOPO TA Cloning Kit from Invitrogen/Life Technologies, USA) [1]. The resulting recombinant vectors were transformed into chemically competent *Escherichia coli* TOP10 cells (also provided by the TOPO TA Cloning Kit). Several positive transformants were analyzed by colony PCR using a combination of a vector-specific primer (M13 forward primer or M13 reverse primer) and an aptamer-specific primer (e.g., primer AP10). This method enables a fast screening for correct plasmid inserts directly from *E. coli* colonies. The plasmid DNA of 96 clones was isolated using the QIAprep 96 Turbo Miniprep Kit from QIAgen (Germany) and the inserted aptamer DNA of each clone was sequenced (Microsynth, Switzerland). The obtained 88 sequences were analyzed and aligned by using the web based tool ClustalW provided by the EBI web server (http://www.ebi.ac.uk/Tools/msa/clustalw2/) [4, 5]. The secondary structure analysis was performed by means of the free-energy minimization algorithm according to Zuker [6] using the internet tool mfold (http://mfold.rna.albany.edu/?q=mfold) [7, 8].

References

1. Stoltenburg R, Reinemann C, Strehlitz B. FluMag-SELEX as an advantageous method for DNA aptamer selection. Anal Bioanal Chem. 2005; 383(1):83-91.

2. Gaillard C, Strauss F. Ethanol precipitation of DNA with linear polyacrylamide as carrier. Nucleic Acids Res. 1990; 18(2):378.

3. Williams KP, Bartel DP. PCR product with strands of unequal length. Nucleic Acids Res. 1995; 23(20):4220-4221.

4. Goujon M, McWilliam H, Li W, Valentin F, Squizzato S, Paern J, et al. A new bioinformatics analysis tools framework at EMBL-EBI. Nucleic Acids Res. 2010; 38(Web Server issue):W695-699.

5. Larkin MA, Blackshields G, Brown NP, Chenna R, McGettigan PA, McWilliam H, et al. Clustal W and Clustal X version 2.0. Bioinformatics. 2007; 23(21):2947-2948.

6. Zuker M. Mfold web server for nucleic acid folding and hybridization prediction. Nucleic Acids Res. 2003; 31(13):3406-3415.

7. Peyret N. Prediction of nucleic acid hybridization: parameters and algorithms [PhD Thesis]. Detroit, MI: Wayne State University, Department of Chemistry; 2000.

8. SantaLucia J, Jr. A unified view of polymer, dumbbell, and oligonucleotide DNA nearest-neighbor thermodynamics. Proc Natl Acad Sci USA. 1998; 95(4):1460-1465.
